# Bacterial glycosyltransferase-mediated cell-surface chemoenzymatic glycan modification

**DOI:** 10.1038/s41467-019-09608-w

**Published:** 2019-04-17

**Authors:** Senlian Hong, Yujie Shi, Nicholas C. Wu, Geramie Grande, Lacey Douthit, Hua Wang, Wen Zhou, K. Barry Sharpless, Ian A. Wilson, Jia Xie, Peng Wu

**Affiliations:** 1 Department of Molecular Medicine, The Scripps Research Institute, La Jolla, CA 92037 USA; 2 Department of Integrative Structural and Computational Biology, The Scripps Research Institute, La Jolla, CA 92037 USA; 3 Department of Chemistry, The Scripps Research Institute, La Jolla, CA 92037 USA; 4 College of Chemistry and Molecular Engineering, Peking University, 100871 Beijing, China; 5 Skaggs Institute for Chemical Biology, The Scripps Research Institute, La Jolla, CA 92037 USA

**Keywords:** Polysaccharides, Chemical modification, Screening

## Abstract

Chemoenzymatic modification of cell-surface glycan structures has emerged as a complementary approach to metabolic oligosaccharide engineering. Here, we identify *Pasteurella multocida* α2-3-sialyltransferase M144D mutant, *Photobacterium damsela* α2-6-sialyltransferase, and *Helicobacter mustelae* α1-2-fucosyltransferase, as efficient tools for live-cell glycan modification. Combining these enzymes with *Helicobacter pylori* α1-3-fucosyltransferase, we develop a host-cell-based assay to probe glycan-mediated influenza A virus (IAV) infection including wild-type and mutant strains of H1N1 and H3N2 subtypes. At high NeuAcα2-6-Gal levels, the IAV-induced host-cell death is positively correlated with haemagglutinin (HA) binding affinity to NeuAcα2-6-Gal. Remarkably, an increment of host-cell-surface sialyl Lewis X (sLe^X^) exacerbates the killing by several wild-type IAV strains and a previously engineered mutant HK68-MTA. Structural alignment of HAs from HK68 and HK68-MTA suggests formation of a putative hydrogen bond between Trp222 of HA-HK68-MTA and the C-4 hydroxyl group of the α1-3-linked fucose of sLe^X^, which may account for the enhanced host cell killing of that mutant.

## Abstract (web summary)

Glycan molecules can be modified directly on the cell surface via chemoenzymatic approaches. Here, the authors employ a set of four bacterial glycosyltransferases to develop a live cell-based killing assay to probe host cell glycan-mediated influenza A virus infection.

## Introduction

Complementary to metabolic oligosaccharide engineering (MOE)^1^, chemoenzymatic glycan labeling and modification have emerged as valuable tools to modify glycan structures within a cellular environment^2–4^. Unlike MOE, which relies on a cell or an organisms’s own glycan biosynthetic mechinary to incorporate unnatural monosaccharides with linkage promiscuity, chemoenzymatic glycan modification utilizes a recombinant glycosyltransferase to transfer natural or unnatural monosaccharides with novel functions from activated nucleotide sugars to glycoconjugates on the cell surface with linkage specificity. For these reasons, chemoenzymatic glycan modification provides a facile and more precise way for probing the function of glycans in cellular processes.

In their pioneering work, Sackstein, Xia et al., applied chemoenzymatic glycan modification based on human α1-3-fucosyltransferase (FT) to install α1-3-linked fucose (Fuc) onto the cell-surface, thereby creating E-selectin ligand, sLe^X^ (NeuAcα2-3-Galβ1-4-(Fucα1-3)-Glc*N*Ac), so as to enhance the engraftment and trafficking of human multipotent mesenchymal stromal cells and cord blood cells^5,6^. In our previous work, we employed chemoenzymatic glycan modification to tune cell-surface receptor signaling and stem cell proliferation^2,7^. Combining this method with bioorthogonal click chemistry, several labs, including our own, have demonstrated that imaging and profiling of specific cellular glycans can be realized^8–10^. Recently, we also applied this method to construct live cell-based glycan arrays on the surface of Chinese hamster ovary (CHO) Lec2 mutant cells possessing a relatively homogeneous repertoire of *N*-linked glycoforms^11^.

To date, glycosyltransferases from both mammalian organisms and bacteria have been used for chemoenzymatic glycan modification. Mammalian Golgi glycosyltransferases are type II transmembrane proteins^12^. For cell-surface glycan modification, truncated versions are often used, including human FT6 and FT7 and ST6Gal1, ST3Gal4, and ST3Gal1^2,5,9,13^. Bacterial glycosyltransferases, on the other hand, often lack the transmembrane domain and, therefore, are more easily expressed in *Escherichia coli* as soluble proteins. Notable examples include *Helicobacter pylori* α1-3-FT (Hp1,3FT), the bacterial homologue of the human blood group A antigen glycosyltransferase, and the *Campylobacter jejuni* β1-4-*N*-acetylgalactosaminyl transferase^10,14,15^. Unfortunately, many bacterial glycosyltransferases that are active for assembly of oligosaccharides in test tubes do not exhibit activities on the cell surface.

**Fig. 1:**
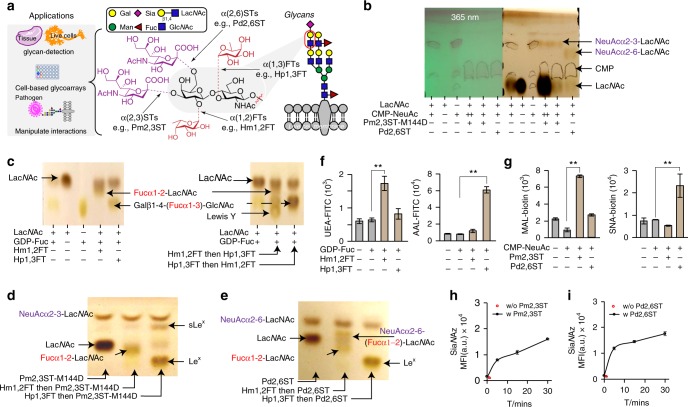
Recombinant bacterial FTs and STs for live cell-surface glycan modification. **a** Specific positions on mammalian cell-surface Lac*N*Ac(Galβ1-4-Glc*N*Ac)-containing glycans that can potentially be modified by fucosylation (α1-2- or α1-3-linked) and sialylation (α2-3- or α2-6-linked). Recombinant bacterial glycosyltransferases (FTs and STs) used in this study include Hm1,2FT, Hp1,3FT, Pm2,3ST-M144D, and Pd2,6ST. **b** Analysis of in vitro sialylation products by TLC. ++ indicates the final reaction system was further mixed with starting material Lac*N*Ac, and analyzed by TLC. **c** Analysis of in vitro fucosylation products by TLC. **d**, **e** Analysis of in vitro products generated by a combination of sialylation and fucosylation by TLC. sLe^X^ was formed by combining Hp1,3FT and Pm2,3ST-M144D (**d**). NeuAcα2-6-(Fucα1-2)-Lac*N*Ac was formed by combining Hm1,2FT and Pm2,3ST-M144D (**e**). **f**, **g** Analysis of newly formed glycan epitopes on the cell-surface of Lec2 CHO cells via chemoenzymatic glycan modification. Modified cells were stained by lectins and analyzed by flow cytometry. **h**, **i** Evaluation of the substrate tolerance of bacterial sialyltransferases. Unnatural sugar CMP-Sia*N*Az bearing the azide group were tested for STs. In figures **f**–**i**, error bars represent the standard deviation of three biological replicates. ** indicated Welch’s *t*-test *P* < 0.01. Source data for figures **b**–**i** are provided as a Source Data file

Here, to expand the enzyme repertoire for chemoenzymatic glycan modification, we perform a screen to identify bacterial glycosyltransferases with relaxed donor specificity that can be used for cell-surface glycan modification. We report that *Pasteurella multocida* α2-3-ST M144D mutant (Pm2,3ST-M144D), *Photobacterium damsela* α2-6-ST (Pd2,6ST), and *H. mustelae* α1-2-FT (Hm1,2FT) can be adopted as useful tools for this application (Fig. 1a). Moreover, Pm2,3ST-M144D and Pd2,6ST are tolerant to large substituents introduced to the C-5 position of the cytidine-5′-monophosphate-*N*-acetylneuraminic acid (CMP-NeuAc) donor. We successfully use these two STs to survey the expression patterns of their respective glycan acceptors in tissue specimens. Combining these enzymes with our previously discovered Hp1,3FT, we develop a live cell-based assay to analyze host-cell glycan-mediated influenza virus infection.

## Results

### Screening recombinant bacterial STs and FTs

In the screening that we performed, natural and unnatural nucleotide sugars functionalized with biotin were used to assess if they can be accepted as the donor substrates of glycosyltransferases of interest. Initially, we focused our screen on sialyltransferases and fucosyltransferases due to the fact that sialic acid (Sia), Fuc, and galactose (Gal) are the three most common monosaccharides found on cell-surface glycans^16^. Sia α2-3- or α2-6-linked to terminal Gal, respectively, are exploited by avian and human influenza virus as receptors for host infection^17^. On the other hand, Fuc residues, when attached to terminal Gal in an α1-2-linkage or attached to the Glc*N*Ac of *N*-acetyllactosamine in an α1-3-linkage, form blood group H antigen and Lewis X (Le^X^, Galβ1-4-(Fucα1-3)-Glc*N*Ac), respectively^16^. Unlike Hp1,3FT, which has been used extensively for glycan modification, no other bacterial STs or FTs have been exploited to transfer biophysical probes (e.g., biotin and fluorescent dyes) directly onto cell surfaces for such an application.

We employed the CHO cell mutant Lec2 cells^18,19^ in this screen. Lec2 has a minimum level of sialylation, resulting in un-capped Lac*N*Ac and polyLac*N*Ac on cell-surface *N*-glycans. After incubating with each individual of these enzymes/donor substrate pair, newly formed glycan epitopes of specific linkage were probed by fluorescently labeled lectins, including *Ulex europaeus* agglutinin 1 (UEA 1, specific for α1-2-linked Fuc), *Aleuria aurantia* lectin (AAL, specific for α1-3- and α1-6-linked Fuc), *Maackia amurensis* lectin (MAL, specific for α2-3-linked Sia, and *Sambucus nigra* lectin (SNA, specific for α2-6-linked Sia). Quantifying the cell-surface lectin staining signals, we discovered two sialyltransferases, Pm2,3ST-M144D^20^ and Pd2,6ST^20–22^, and a fucosyltransferase, Hm1,2FT^23^, that can install natural sialic acid or fucose, respectively, onto the cell surface (Figs. 1f, g). Consistent with our previous observations, robust AAL staining siganl was obtained when Lec2 cells were treated with Hp1,3 FT and guanosine 5’-diphospho-Fuc (GDP-Fuc). For enzymes providing positive signals, dose-dependent modification was observed. For example, as shown in Supplementary Fig. 2, cell-associated ECA staining decreased while SNA staining increased along with increasing the concentration of the CMP-NeuAc for Pd2,6ST-mediated Lec2 cell sialylation.

To further validate the activities of Hm1,2FT, Hp1,3FT, Pm2,3ST-M144D, and Pd2,6ST, we performed in vitro glycosylation reactions using the natural donor substrates, CMP-NeuAc (for STs) and GDP-Fuc (for FTs), and type 2 *N*-acetyllactosamine (Lac*N*Ac, Galβ1-4-Glc*N*Ac) as the acceptor. Thin-layer chromatography (TLC) and liquid chromatography-mass spectrometry (LC/MS) analysis confirmed the formation of Fucα1-2-Galβ1-4-Glc*N*Ac, NeuAcα2-3-Galβ1-4-Glc*N*Ac, NeuAcα2-6-Galβ1-4-Glc*N*Ac, and Le^X^ in Hm1,2FT, Pm2,3ST-M144D, Pd2,6ST, and Hp1,3FT-mediated transformations, respectively (Figs. 1b, c). Consistent with a previous report^22^, when trimeric Lac*N*Ac was used as the acceptor substrate, both terminal and internal galactose residues were modified by Pd2,6ST.

Subsequently, Fucα1-2-Galβ1-4-Glc*N*Ac was treated with Hp1,3FT or Pd2,6ST to produce Lewis Y or Fucα1-2-(NeuAcα2-6)-Galβ1-4-Glc*N*Ac, respectively (Figs. 1c–e). By treating Le^X^ with Pm2,3ST-M144D and CMP-NeuAc, sLe^X^ was produced (Fig. 1d).

### Evaluating the donor substrate promiscuity of STs and FTs

Originally reported by Chen and coworkers, Pm2,3ST-M144D and Pd2,6ST, are highly efficient for one-pot chemoenzymatic oligosaccharide synthesis^20,24^. These enzymes have broad substrate scopes, tolerating functional groups including azide, alkyne, acetyl, O-methyl introduced at either the *N*-acyl side chain or the C-9 position. Likewise, Hm1,2FT has been used to synthsize the human blood H antigen. However, the donor substrate scope of this enzyme remains unexplored. To profile the tolerance of the above three enzymes for unnatural donor substrates to modify cell-surface glycans, we used azide-bearing sugar donors GDP-FucAz and CMP-Sia*N*Az. In this experiment, Lec2 cells were incubated with a sialyltransferase (Pm2,3ST-M144D or Pd2,6ST) and CMP-Sia*N*Az, or with a fucosyltransferase (Hp1,3FT or Hm1,2FT) and GDP-FucAz. Following the enzymatic treatment, the modified cells were reacted with an alkynyl biotin via the ligand (BTTPS)-assisted copper-catalyzed azide-alkyne [3 + 2] cycloaddition reaction (CuAAC)^25^, and probed with Alexa Fluor 488-streptavidin. Flow cytometry analysis revealed that Pm2,3ST-M144D- or Pd2,6ST-treated Lec2 cells were robustly labeled, and the labeling was time-dependent (Figs. 1h, i). As expected, Hp1,3FT-treated cells also exhibited significant labeling (Supplementary Fig. 3A, B). However, no signals were detectable for the Hm1,2FT-treated cells (Supplementary Fig. 3C), suggesting that this enzyme is unable to accept the azide-functionalized donor. The non-tolerance of unnatural donors by Hm1,2FT was further confirmed by in vitro Lac*N*Ac modification (Supplementary Fig. 3D, E).

Further evaluation of the donor substrate scope of Pm2,3ST-M144D and Pd2,6ST revealed that besides the *N*-acyl modified CMP-Sia*N*Az, these two enzymes were capable of incorporating other CMP-Sia analogs, including CMP-9AzSia^26^, CMP-Sia*N*Al^27^, and CMP-Sia*N*Poc^11^, onto cell-surface glycans (Supplementary Fig. 4).

**Fig. 2:**
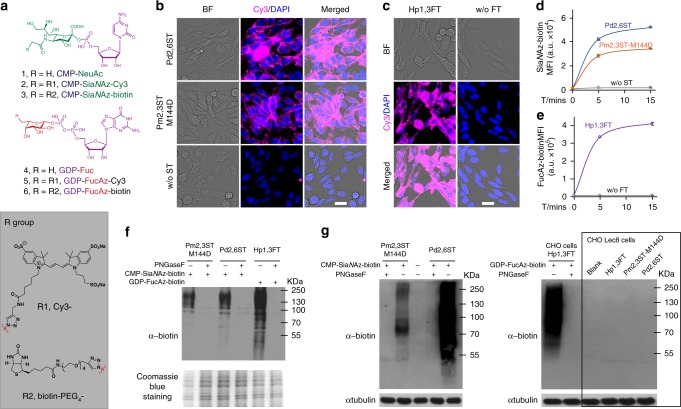
One-step glycan labeling enabled by recombinant bacterial glycosyltransferases. The Pm2,3ST-M144D, Pd2,6ST, or Hp1,3ST-mediated incorporation of unnatural sugars conjugated to a fluorescent dye (Cy3) or an affinity tag (biotin), enabled a One-step cell-surface glycan labeling. **a** Nucleotides and analogs functionalized with biotin tag (CMP-Sia*N*Az-biotin and GDP-FucAz-biotin) or with Cy3 florescent dye (CMP-Sia*N*Az-Cy3 and GDP-FucAz-Cy3). **b** Direct STs-catalyzed conjugation of Cy3 (magenta) for imaging of live cell glycans. **c** Hp1,3FT-catalyzed conjugation of Cy3 (magenta) for imaging of live cell glycans. In **b** and **c**, cells were visualized by bright field images and DAPI staining (blue). Scale bar, 20 μm. **d** Time-dependence of activities of recombinant bacterial and human STs for cell-surface glycan labeling with CMP-Sia*N*Az-biotin. **e** Activity of Hp1,3FT using GDP-FucAz-biotin to conjugate biotin onto live cell-surface glycan directly. In **d** and **e**, error bars represent the standard deviation of three biological replicates. **f**, **g** Enzyme-assisted incorporation of biotin was mainly on *N*-linked glycans on CHO cells and CHO Lec2 cells, while CHO mutant Lec8 cells without Lac*N*Ac were not labeled. Protein loading was depicted by Coomassie blue staining or anti-tubulin western blot. Source data for figures **d**–**g** are provided as a Source Data file

To survey if the promiscuity of Pm2,3ST-M144D and Pd2,6ST could enable the transfer of biotin- or Cy3-functionalized CMP-Sia derivatives directly to the cell surface for one-step glycan labeling, Lec2 cells were incubated with either enzyme in the presence of crude conjugation product of CMP-Sia*N*Az-Cy3 or CMP-Sia*N*Az-biotin. Biotinylated cells were further probed with Alexa Fluor 647-streptavidin. The cell-surface fluorescence of streptavidin-labeled or Cy3-labeled cells were then quantified by flow cytometry or examined by fluorescence microscopy, respectively. We detected strong fluorescent signal in both enzyme-treated cells. In control experiments, only background fluorescence was observed for cells treated with CMP-donors in the absence of both STs (Fig. 2a–d). To confirm that these signals were produced from glycoprotein labeling, lysates of treated Lec2 cells, CHO cells and Lec8 cells were collected. Anti-biotin western blot confirmed that biotin was incorporated into glycoproteins of Lec2 cells and CHO cells (MW 55-250 KD), not the mutant CHO Lec8 cells that lack cell-surface Lac*N*Ac (Fig. 2f, g). Moreover, PNGase F releasement of *N*-linked glycans essentially abolished all signal of labeled CHO and Lec2 cells, suggesting that Lac*N*Ac residues in *N*-linked glycans are the primary targets labeled by these enzymes. However, it is also possible that CHO cells express low levels of extended core 1 and core 2 *O*-glycans. Therefore, there are few acceptor substrates to be modified by ST(Pm2,3ST-M144D or Pd2,6ST).

### STs-based chemoenzymatic labeling of tissue specimens

**Fig. 3:**
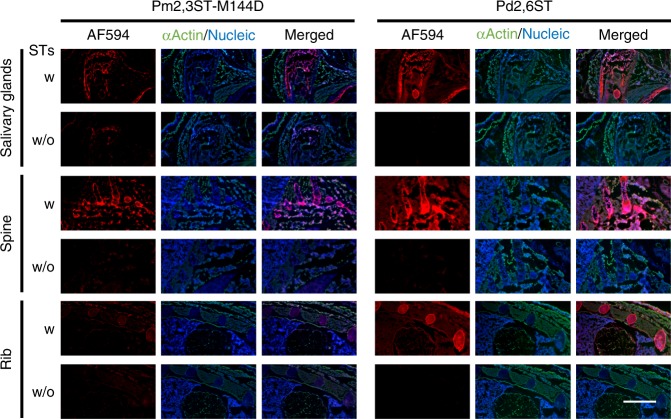
One-step recombinant bacterial STs-based labeling of glycans in tissue specimens. The embryonic frozen sections from E16 mouse were incubated with STs (Pm2,3ST-M144D or Pd2,6ST) or without STs, and CMP-Sia*N*Az-biotin, followed by Alexa Fluor 594-streptavidin conjugate staining. The resulting fluorescence (red) of different parts of the embryo was directly imaged using microscopy, including salivary glands region, lateral sections of spine, and anterior chest. The cells of frozen sections were stained with anti-actin (green) and DAPI (blue, nucleus). Scale bar, 1 mm

Next, we evaluated the feasibility of labeling tissue specimens via one-step ST(Pm2,3ST-M144D or Pd2,6ST)-mediated glycan modification. Whole embryo frozen sections from C57BL/6 mouse (E16) were incubated with either STs and CMP-Sia*N*Az-biotin before staining with Alexa Fluor 594-streptavidin and imaged using fluorescence microscopy. In contrast to the higher background of the traditional two-step strategy using azide-bearing unnatural sugars followed by CuAAC-conjugation of biotin, Pd2,6ST-mediated one-step tissue glycan labeling showed much better contrast (Supplementary Fig. 5). Interestingly, compared to samples without enzyme-treatments, tissue slides treated with STs showed robust fluorescence with distinct labeling patterns (Fig. 3, and Supplementary Fig. 6, 7). The outer skin and the salivary gland region exhibited intensive signals afforded by labeling with both enzymes. Interestingly, Pd2,6ST-labeling generated significantly higher signals than Pm2,3ST-M144D-labeling in bone structures, including the sections of leg, rib, spine, and skull. When tissue sections were digested first with PNGase F to remove *N*-glycans before incubating with either STs, CMP-Sia*N*Az-biotin, and Alexa Fluor 594-streptavidin, Alexa Fluor 594-associated fluorescence was still detectable in most organs, strongly suggesting that other glycoconjugates (e.g., *O*-glycans) are also labeled (Supplementary Fig. 7).

### Probing IAV-HA-glycan interactions via live cell-based array

**Fig. 4:**
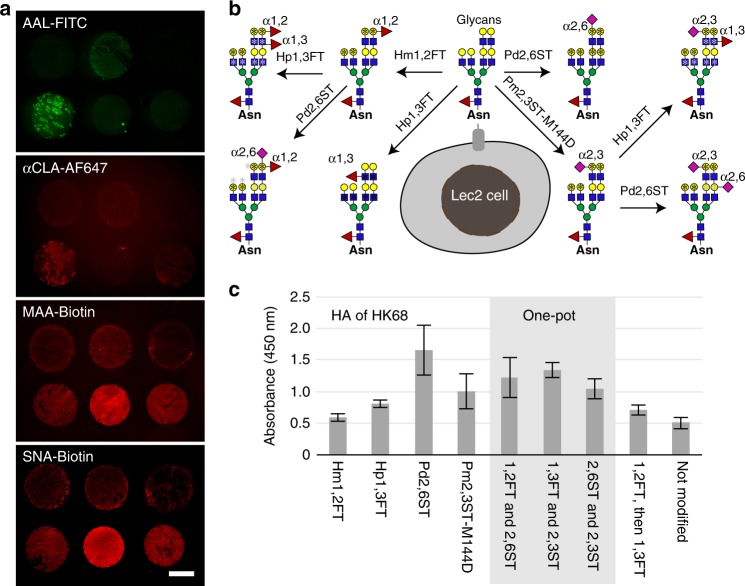
A cell-based glycan array to assess HA–glycan interactions directly on live cells. **a** Profiling glycoforms of lung tissues obtained from healthy human donors. Lung tissue slides were stained with FITC-AAL, AF647-anti-CLA, Biotin-MAA, or Biotin-SNA conjugates to detect α1-3-fucosylation, sLeX epitopes, α2-3-linked, or α2-6-linked sialylation, respectively. **b** Major glycan epitopes presented on Lec2 cell-surface after chemoenzymatic glycan modification. CHO Lec2 cells were treated with glycosyltransferases indicted above and the corresponding nucleotide sugars. *indicates the potential modification site for the first-step glycan modification (black), and the second-step glycan modification (gray). **c** Relative binding affinity of HA from HK68 (H3N2) for glycan-modified Lec2 cells using the specified recombinant glycosyltransferases. In Fig. 4c, the error bars represent the standard deviation of six biological replicates. Source data for figure **c** are provided as a Source Data file

As another application of chemoenzymatic cell-surface glycan modification, we probed how changes to live host-cell glycosylation patterns impact IAV infection. The attachment of the HA of IAV to the sialylated glycans of host epithelium is the first step in the viral entry cycle^17^. Glycan microarrays have been heavily employed to identify sialylated glycoepitopes that act as host receptors for IAV and to uncover the Sia binding-preferences of different HAs or whole viruses^28–33^. It has been found that human IAVs prefer NeuAcα2-6-linked to Gal (human-type), which is abundantly expressed on epithelial cells of the human airway. By contrast, avian IAVs prefer NeuAcα2-3-Gal (avian-type) and bind poorly to the human upper airway epithelium^34–37^. Despite the rich information gleaned from glycan microarray-based analyses, our understanding of HA-glycan interactions is incomplete without elucidating its physiological relevance. The solid-phase based glycan arrays do not capture the entire potential diversity of glycans present on the cell surface. As revealed by the lectin staining of lung tissues from different donors, cell-surface glycosylation patterns vary from individual to individual, exhibiting fluctuations in α2-3- or α2-6-linked sialylation, α1-3-fucosylation, and sLe^X^ expression (Fig. 4a, and Supplementary Fig. 8). The variation of glycan expression in a person’s respiratory tract may possibly account for differential susceptibility to influenza infection. We hypothesize that creating specific glycan epitopes that were previously identified by microarray-based binding assays directly on the live cell surface may serve as a quick way to dissect their specific contributions in a more native environment.

Currently, only two influenza A subtypes circulate within humans: namely H1N1 and H3N2^31^. Using Lec2 cells and a combination of the four enzymes described above, we assembled a small cell-based glycan array (Fig. 4b). We incubated the HA of influenza A/HongKong/1/1968 (HK68, H3N2) with this array and assessed its binding preference. As expected, HA of HK68 exhibited strong preference for NeuAcα2-6-linked to Gal. Surprisingly, it also exhibited significant binding with sLe^X^ created by 1,3FT and 2,3 ST on the cell surface (Fig. 4c).

### Studying IAV infection in glycan-modified host cells

To evaluate if the interaction with sLe^X^ on the host-cell surface plays any role in the viral infection, we adopted a live cell-based infection assay, in which we in situ edited the glycocalyx of Madin–Darby canine kidney (MDCK) cells, a well-established cell line for studying IAV, using the aforementioned glycosyltransferases. Cultures of the glycocalyx-modified cells, untreated cells, or cells treated with nucleotide sugars only were infected with serial dilutions of IAV in 96-well plates. This assay provides a direct approach to evaluate the impact of Sia and Fuc that are attached to the cell surface with distinct linkages on the susceptibility of host cells to IAV infection, enabling correlating glycosylation patterns with host-cell killing.

**Fig. 5:**
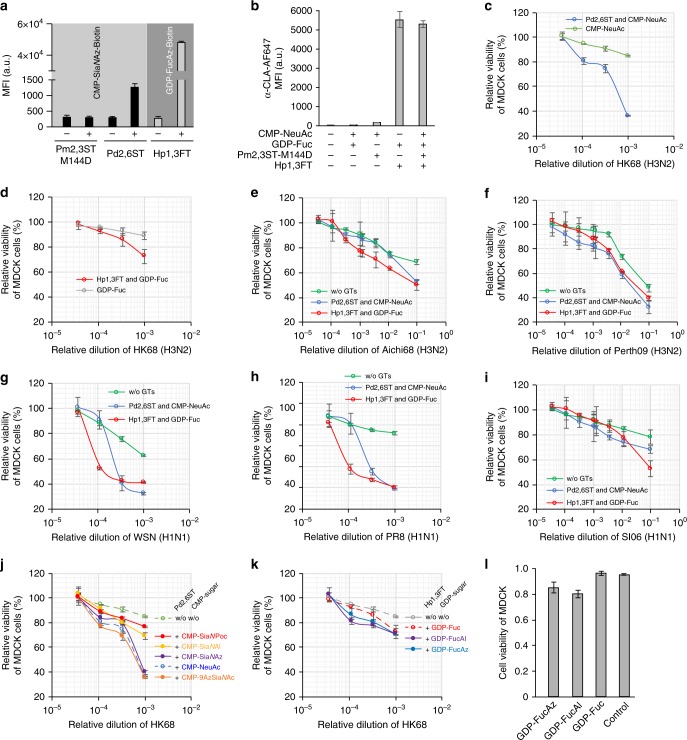
Profiling IAV infection using glycocalyx-modified MDCK cells. **a** Modification of glycocalyx of MDCK cells using Pm2,3ST-M144D, Pd2,6ST, or Hp1,3ST and the corresponding donor substrate conjugated with biotin. Biotinylated cells were probed with Alexa Fluor 647-Streptavidin. **b** Modification of glycocalyx of MDCK cells using a combination of Pm2,3ST-M144D and Hp1,3ST. Newly generated sLe^X^ on the MDCK cell surface was confirmed by Alexa Fluor 647-anti-CLA conjugate staining. **c** Viability of Sia-edited MDCK cells or control cells upon infection by HK68. **d** Viability of Fuc-edited MDCK cells or control cells infected by HK68. **e**–**i** Viability of glycan (Sia or Fuc) edited MDCK cells or control cells upon infection by Aichi68 (**e**), Perth09 (**f**), WSN (**g**), PR8 (**h**), and SI06 viruses. **j**–**l** Viability of glycan edited MDCK cells or control cells upon infection by HK68, using analogs of CMP-Sia (**j**) or GDP-Fuc (**k**). Viability of Fuc-edited MDCK cells or control cells, at 10^−4^ virus dilution (**l**). In figures **a** and **b**, the error bars represent the standard deviation of three biological replicates. In **c**–**l**, the error bars represent the standard deviation of six biological replicates. Source data are provided as a Source Data file

As found previously, both NeuAcα2-6-Gal and NeuAcα2-3-Gal are present on the surface of MDCK cells^38^. However, the expression level of NeuAcα2-6-Gal is low^39^. Using Pd2,6ST, additional NeuAcα2-6-Gal epitopes can be created by adding NeuAc to the terminal and internal Gal residues of the Lac*N*Ac repeats (Fig. 5a and Supplementary Fig. 9B). This is confirmed by the increase in the SNA staining, which reached a plateau when ~250 μM CMP-NeuAc was used (Supplementary Fig. 10A). Likewise, using Hp1,3 FT- mediated in situ Fuc modification, sLe^X^ epitopes can be readily created as confirmed by the anti-cutaneous lymphocyte-associated antigen (anti-CLA) immunostaining (Fig. 5b). To futher characterize the newly created glycan epitopes on the cell surface, we performed MALDI-TOF analysis of *N*-linked glycans of the Sia-edited and Fuc-edited cells. As shown in Supplementary Fig. 11, the appearence of a tetra-antennary *N*-glycan with four sialic acids added to the peripheral galactose was clearly identified in Sia-edited cells, but was not detectable in untreated MDCK cells. In addition, the peak intensity corresonding to sialylated bi-antennary epitopes (designated with asterisk in Supplementary Figs. 11B and 11C) also increased significantly. Likewise, the newly created bi-antennary, tri-antennary, and tetra-antennary sLe^X^ epitopes, as well as a tetra-antennary Le^X^, were found in Fuc-edited cells.

Naturally occurring H3N2 strains, HK68, A/Aichi/2/1968 (Aichi68) and A/Perth/16/2009 (Perth09), and an H1N1 strain, A/Solomon Islands/3/2006 (SI06), as well as two laboratory-derived H1N1 strains, A/WSN/33 (WSN) and A/Puerto Rico/8/1934 (PR8), were used in this infection assay. We first subjected MDCK cells to Pd2,6ST-mediated or FT-mediated modification to increase cell-surface NeuAcα2-6-Gal epitopes or create new sLe^X^ epitopes, respectively. Next, we incubated the modified cells or cells treated with nucleotide sugars only with serial dilutions of IAV. Two days later, the host-cell viability was analyzed.

As expected, increasing NeuAcα2-6-Gal epitopes enhanced IAV-dependent cell killing for all strains tested, especially at high viral titers (Fig. 5). As shown in Supplementary Fig. 9B, the higher the concentration of CMP-NeuAc that was used to install NeuAc onto the cell surface, the more severe the HK68-induced killing became. It was clearly observed that the killing reached a plateau at 10^−5^ and 10^–4^ viral dilutions when ~250 μM CMP-NeuAc was used, which is consistent with the maximum amounts of NeuAc residues that can be installed on MDCK cells. In the control experiment, treating cells with the donor substrate CMP-NeuAc but without Pd2,6ST only had a minor impact on viral infection (Figs. 5c–i). Interestingly, the newly added sLe^X^ epitopes on the host-cell surface also augmented influenza-induced cell death (Figs. 5d–i). At 10^–1^ viral dilution, 50 ± 4% (±, the standard deviation of six biological replicates), 39 ± 2%, 53 ± 6% of the sLe^x^ decorated cells remained viable upon incubating with Aichi68, Perth09 or SI06 (H1N1), respectively. By contrast, 68 ± 2%, 48 ± 3%, 78 ± 6% of the unmodified cells were viable upon infection by these viral strains. More pronounced effects induced by the sLe^x^ addition were observed upon infection with HK68, WSN, or PR8; at 10^–3^ viral dilution, only 73 ± 6%, 40 ± 1%, 41 ± 1% of the infected cells that were modified by sLe^x^ remained viable, respectively, whereas 89 ± 3%, 62 ± 1%, 92 ± 2% of the unmodified cells were viable following infection by these viral strains. The fucosyltransferease-mediated sLe^X^ creation also induced dose-dependent host-cell killing upon IAV infection. As shown in Supplementary Fig. 12 for the infection with WSN, at a viral dilution of 10^−3^–10^−5^, the maximum killing was achieved when ~100 μM GDP-Fuc was used (Supplementary Fig. 12B), which is consistent with the maximum quantities of sLe^X^ epitopes that can be created on MDCK cells (Supplementary Fig. 12A).

MDCK cells modified by unnatural Sia and Fuc analogs were also evaluated in this infection assay using HK68. As shown in Fig. 5j, although C-9- and *N*-acetyl-Az modified Sia α2-6-linked to Gal exhibited similar activities as the natural ones to promote the influenza virus infection, α2-6-linked Sia*N*Al and Sia*N*Poc installed via the same fashion showed reduced activities (Fig. 5j and Supplementary Fig. 13). Finally, all Fuc analogs examined were found to share similar functions at 10^−3^ virus titer. However, at 10^−4^ virus titer, the alkyne-bearing fucose analog, FucAl, seemed to enhance host-cell infection by HK68 (Fig. 5k, l).

### Profiling the structural constraints of HA for glycan binding

Then, the impacts of different HA structures on the binding of host-cell-surface glycan were profiled via chemoenzymatic glycan modification. H3N2 IAV have circulated in humans since 1968, but antigenic drift of HA continues to be a driving force that enables the virus to escape prior immunity. Since the major antigenic sites of the HA overlap with the receptor-binding site (RBS), the virus constantly evolves to effectively adapt to host immune responses without compromising its virulence^40–42^. The RBS consists of the 130-loop, 150- loop, 190-helix, and 220-loop (Wilson et al., 1981)^43^. While the 130-loop, 150-loop, and 190-helix are relatively conserved among HA subtypes, a higher genetic diversity has been detected in the 220-loop, which reflects also some differences in residues responsible for receptor specificity in the different subtypes (e.g., H1N1 vs. H3N2)^29,30^.

To examine if sequence variation within the HA-RBS confers H3N2 influenza viruses any advantage to infect host cells harboring NeuAcα2-6-Gal epitopes or sLe^X^ epitopes, we further assessed the wild-type HK68 virus and three laboratory-derived 220-loop mutants that can potentially escape from preexisting immunity, although exhibiting weaker binding toward the NeuAcα2-6-Gal receptor^41^. HK68-MTA (G225M/L226T/ S228A), HK68-LSS (G225L/L226S), and HK68-QAS (G225Q/ L226A) share a very similar HA backbone conformation, but their binding affinity for NeuAcα2-6-Gal decreases following the order of HK68-MTA > HK68-LSS > HK68-QAS^41^. All three mutants were found to have WT-like virus replication fitness in unmodified MDCK cells presumably due to the low level of α2-6-linked Sia expressed in this cell line.

**Fig. 6:**
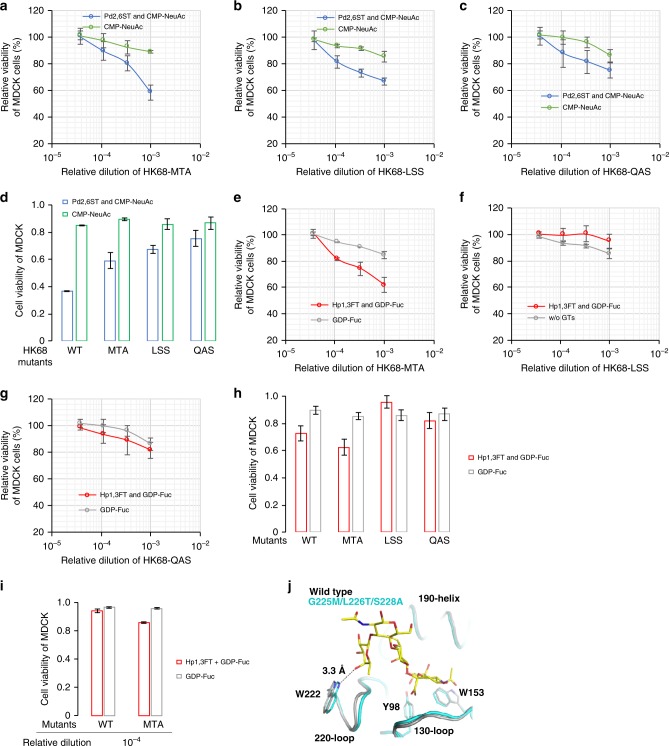
Profiling the structural constraints of IAV-HA for glycan binding. The activities of wild-type HK68 and its hemagglutinin-receptor-binding site mutants to infect Sia- or Fuc-edited host cells, were directly compared via host-cell killing. **a**–**d** Viability of Sia-edited MDCK cells or control cells upon infection by wild-type HK68 and its HA-RBS mutants, including HK68-MTA (**a**), HK68-LSS (**b**) and HK68-QAS (**c**). **d** Cell viability at 10^−3^ viral dilution. **e**–**i** Viability of Fuc-edited MDCK cells or control cells upon infection by wild-type HK68 and its HA-RBS mutants. Cell viability at 10^−3^ virus dilution (**h**), and at 10^−^^4^ virus dilution (**i**). **j** Structural alignment of HAs from HK68 and HK68-MTA. A minor shift of 220-loop backbone of HK68-MTA enables formation of a H-bond between C4 hydroxyl of α1-3-linked fucose of sLe^x^ and Nε1 of W222 (Fig. 6I), which is not observed between the HK68-WT HA and sLe^X^. In Fig. 6A-I, the error bars represent the standard deviation of six biological replicates. Source data for figures **a**–**i** are provided as a Source Data file

To evaluate viral infection in MDCK cells harboring elevated NeuAcα2-6-Gal epitopes or sLe^X^ epitopes, we treated the glycan-modified cells with WT HK68 or the three mutants. Consistent with previous observations^41^, all four strains exhibited similar host-cell killing capabilities in unmodified MDCK cells (Figs. 6a–d). By contrast, upon elevating the cell-surface NeuAcα2-6-Gal levels, the capability to induce host-cell death compared to wild-type HK68 was observed to be HK68-MTA > HK68-LSS > HK68-QAS, which matched their NeuAcα2-6-Gal binding affinities. Interestingly, these same mutants manifested different killing capabilities in host cells harboring sLe^X^ epitopes. Compared with WT HK68, enhanced killing was observed for HK68-MTA, whereas HK68-LSS and HK68-QAS exhibited decreased capability to infect sLe^X^-decorated host cells (Figs. 6e–i).

Compared with HK68, HK68-MTA was found to possess better preference for sLe^X^ harboring cells, especially at low viral titers (Fig. 6i). In order to probe the molecular basis for this observation, apo structures of HK68-WT HA (PDB 4FNK)^44^ and HK68-MTA HA (PDB 5VTX)^41^ were aligned with the crystal structure of A/canine/Colorado/17864/06 (H3 subtype)^45^ HA in complex with sLe^X^ using the RBS (residues 117–265 of HA1)^46^. As previously described^41^, a minor shift of 220-loop backbone of 0.8 Å was observed in HK68-MTA HA. Our alignment revealed that this shift likely enabled the formation of an H-bond between the C4 hydroxyl of Fuc and Nε1 of W222 (Fig. 6j), which could not be formed between the HK68-WT HA and sLe^X^. Specifically, the distance between the C4 hydroxyl of Fuc and Nε1 of W222 in HK68-MTA HA is 3.3 Å, which is at the high end of the hydrogen-bond distance range (2.2–3.5 Å). By contrast, the distance between C4 hydroxyl of Fuc and Nε1 of W222 of the wild-type HA is 3.6 Å, which is outside of the normal hydrogen-bond range. This interaction is likely to be responsible for the better binding affinity of HA-HK68-MTA to sLe^X^ and accordingly the enhanced host-cell killing compared with wild-type HK68.

## Discussion

In 1979, Paulson et al. first demonstrated that Sia could be directly transferred from CMP-Sia to the cell surface of desialylated erythrocytes using recombinant mammalian sialyltransferases^4^. Recently, due to the creation of the expression vector library encoding all known human glycosyltransferases by Moremen et al., any human glycosyltransferase of interest can now be produced as secreted catalytic domain GFP-fusion proteins in mammalian and insect cell hosts^47^. Studies by Boons, Steet and coworkers and by our own lab have demonstrated that several enzymes produced by this system are highly efficient for cell-surface chemoenzymatic glycan modification^2,9,48^. However, this approach is associated with relatively high cost. For cell-surface labeling studies, the GFP tag usually needs to be cleaved before treating cells due to non-specific bindings of GFP to the plasma membrane. Complementary to mammalian glycosyltransferases, bacterial counterparts have been developed for cell-surface glycan modification. Significantly, our most recent study have demonstrated that certain bacterial glycosyltransferases, e.g., HpFT, possesses remarkable donor substrate scope such that even DNA or antibody-conjugated nucleotide sugar donors can be recognized and transferred to the cell surface and endow the modified cells with desired functions^49^.

In this study, we discovered that bacteria-derived Pm2,3ST-M144D, Pd2,6ST, and Hm1,2FT can be exploited for cell-surface glycan labeling and modification. As demonstrated previously and also here, these enzymes were easily prepared in multi-milligram quantities in *E. coli* as His-tagged recombinant proteins. Among these three enzymes, Pm2,3ST-M144D and Pd2,6ST were found to tolerate a CMP-Sia donor functionalized with biotin or Cy3, enabling cell-surface acceptor glycans to be tagged with these probes for enrichment or visualization. Applying Pm2,3ST-M144D and Pd2,6ST-mediated chemoenzymatic glycan modification to label whole embryo frozen sections from C57BL/6 mice (E16), we found that the salivary gland expressed high levels of acceptor glycans of both enzymes. Sia was first isolated from bovine submaxillary mucin by Blix in 1936^50^. Thus, it is not surprising that salivary gland expressed high levels of sialyltransferase acceptors. Interestingly, in the developing bones Pd2,6ST-labeling yielded much higher signals than Pm2,3ST-M144D-labeling. Although Pm2,3ST-M144D can only label the terminal Gal, Pd2,6ST is capable of labeling galactoses of internal Lac*N*Ac units^22^. The distinct labeling pattern observed here suggests that abundant polyLac*N*Ac glycans are present in bones and in cartilage. This observation is consistent with a previous report that revealed that polyLac*N*Ac were predominantly found in *N*-glycans of undifferentiated human bone marrow mesenchymal stem cells^51^.

Combined together with our previously discovered *H. pylori* 1,3FT, Pm2,3ST-M144D and Pd2,6ST were used to create a diverse array of sialylated and fucosylated glycan epitopes on the cell surface. By using MDCK cells modified via this enzyme-mediated glycan modification to probe the infection of wild-type HK68 and its HA mutants, we confirmed that the ability of an IAV to induce host-cell death is positively correlated to the Sia*N*Acα2-6-Gal binding affinity of the viral HA. Furthermore, this correlation is dose dependent—only at high levels of cell-surface NeuAcα2-6-Gal can this correlation be observed. Unexpectedly, besides NeuAcα2-6-Gal receptors, several naturally occurring H1N1 and H3N2 strains also recognized sLe^X^ epitopes on the host cells, to facilitate their infection. As is the case for the newly created NeuAcα2-6-Gal epitopes, increasing the quantity of sLe^X^ on the cell surface exacerbates the severity of IAV infection in a dose-dependent manner.

HA is the major surface antigen that evolves at an exceptionally high rate. Variation in the HA-RBS through antigenic drift has produced changes in receptor binding that begins to blur the definition of human-type receptor specificity^42,52–54^. Our investigation uncovered that several H3N2 and H1N1 strains, including Aichi68 (H3N2), WSN (H1N1), and PR8 (H1N1), exhibit preference for high sLe^X^-bearing cells over high Sia*N*Acα2-6-Gal-bearing cells especially at low viral titers (Fig. 5). At low virus dilutions, these strains induced significantly higher levels of cell death in sLe^X^-harboring MDCK cells than in Sia*N*Acα2-6-Gal-harboring counterparts. These observations suggest that such strains may selectively infect human populations with high sLe^X^-expression in their respiratory tracts, such as patients with cystic fibrosis and patients suffering from airway inflammation^55^. It has been documented that several avian influenza virus strains exhibit strong affinities for sLe^X^-type receptors^36,37,56^. Therefore, it is likely that human populations with high sLe^X^-expression in their respiratory tracts are susceptible to these viruses as well.

Our studies strongly suggested that binding specificity and strength to HA are not only encoded in the structure of individual glycans, but also are determined by the density of these epitopes on the cell surface, which is contributed by repeating unit copies that are found in a single glycan or its neighboring structures. This context-dependent molecular recognition underscores the importance of tools that empower the investigation of glycan functions within a more native environment such as the cell surface. The chemoenzymatic glycan modification technique described here should serve as a valuable tool for accomplishing this goal. Currently, we are applying this technique to explore the impact of changes to cell-surface glycosylation patterns on the infection of other types of human viruses.

## Methods

### Enzyme activity assay for donor and acceptor substrates

The activity of purified enzymes was tested by TLC and LC-MS. All reactions were carried out at 37 °C in 40 μL of 50 mM Tris-HCl (pH 7.5) containing 10 mM MgSO_4_. 5 mM *N*-acetyllactosamine (Lac*N*Ac) was used as the acceptor substrate for all enzymes, and 5 mM CMP-NeuAc or GDP-Fucose as the donor. For Hp1,3FT, Pd2,6ST, and Pm2,3ST-M144D, the enzymes were added at a concentration of 0.15 μg/μL, and the reaction time was 30 min. Hm1,2FT was used at 0.3 μg/μL, and the reaction time was 4 h. To further investigate the acceptor specificity of the enzymes, we performed sequential enzymatic reactions, by adding another enzyme and the required donor substrate after completing the current reaction. Lewis X (Le^X^)was produced by fucosylating Lac*N*Ac with Hp1,3FT as described above, and sialyl-Lewis X (sLe^X^) was produced by adding 5 mM CMP-NeuAc and 0.15 μg/μL Pm2,3ST to the reaction. Fucα1-2-(NeuAcα2-6)-Galβ1-4Glc*N*Ac or Lewis Y (Le^Y^) was generated by sequentially adding Hm1,2FT and Pd2,6ST (or Hp1,3FT) reactions, respectively. The donor tolerance tests were performed under the same conditions above using unnatural nucleotide sugar analogs (GDP-FucAz and GDP-FucAl). For TLC analysis, isopropanol: H_2_O: NH_4_OH (8:3:2) was used as the development solvent, the nucleotide sugar was visualized under a 365 nm ultraviolet lamp, while Lac*N*Ac and the products were visualized by staining with 10% sulfuric acid in ethanol. For LC-MS analysis, 100 μL of ethanol was added to the reaction mixture and centrifuged at 13,000 × *g* for 2 min. The supernatant was then analyzed by LC-MS under positive mode (for fucosyltransferases-catalyzed reactions) or negative mode (for sialyltransferases-catalyzed reactions).

### Chemoenzymatic glycan labeling

For flow cytometry, the cultured cells were collected, washed twice with PBS, and resuspended in labeling buffer (HBSS buffer with 3 mM HEPES and 20 mM MgSO_4_). About 150,000 cells were used in a total reaction volume of 50 μL, containing ~3 μg enzyme and 0.2 mM nucleotide sugar donor. In lectin staining, natural GFP-Fucose or CMP-NeuAc was used. After incubating at 37 °C for 15 min, the cells were washed twice and resuspended in 50 μL HBSS buffer containing 10 mM CaCl_2_, 10 mM MgCl_2_, 10 μg/mL FITC (or biotin)- conjugated lectins (AAL-FITC, UEA-FTIC, SNA-biotin, ECA-biotin and MAL-biotin), and 1 in)- conjugated lectin on ice in dark for 30 mins, cells were washed three times and resuspended in 100 μL HBSS buffer containing 10 mM CaCl_2_ and 10 mM MgCl_2_. In one-step glycan labeling of cells, GDP-FucAz-biotin or CMP-Sia*N*Az-biotin was used. After incubating at 37 °C for 15 mins, the cells were washed twice with PBS and resuspended in 50 μL FACS buffer (PBS containing 0.5 mM EDTA and 2% FBS) with 5 μg/mL Alexa Flour 488-streptavidin (or AF647-streptavidin as indicted) and 1 μg/mL DAPI. Then, the cells were kept on ice in dark for 30 min, washed twice and resuspended in 100 μL FACS buffer. In two-step labeling, GDP-FucAz or CMP-Sia*N*Az was used. After incubating at 37 °C for 15 min, the cells were washed twice with PBS and resuspended in 100 μL PBS containing 0.5% FBS, 50 μM CuSO_4_, 300 μM BTTPS, 2.5 mM sodium ascorbate, and 50 μM Alkyne-PEG_4_-biotin. The click reaction was carried out at rt for 10 mins and quenched with 2 μL 50 mM bathocuproine disulfonate (BCS). The cells were then washed twice with PBS and resuspended in 50 μL FACS buffer (PBS containing 0.5 mM EDTA and 2% FBS) with 5 μg/mL Alexa Fluor 488-streptavidin and 1 μg/mL DAPI. The cells were kept on ice in the dark for 30 min, and washed twice and resuspended in 100 μL FACS buffer. The resuspended cells were then analyzed by flow cytometry. The one-step biotin labeling of cell surface Lac*N*Ac containing glycan with Pm2,3ST and Pd2,6ST was performed in different cell lines. After staining  with AF647 (or AF488)-streptavidin conjugates, cell-surface fluorescence was detected by flow cytometry.

For fluorescent imaging, the one-step fluorescent labeling of cell-surface Lac*N*Ac containing glycan with STs (Pm2,3ST or Pd2,6ST) or Hp1,3FT was performed in Lec2 cells using CMP-Sia*N*Az-Cy3 (or GDP-FucAz-Cy3) and imaged by fluorescence microscopy. For one-step biotin labeling of LacNAc containing glycans in tissues, slides were incubated with HBSS (pH 7.4) buffer with 3 mM HEPES, 20 mM MgSO_4_ and 100 μM CMP-Sia*N*Az-biotin, and 0.3 μg/mL enzymes (Pm2,3ST-M144D or Pd2,6ST) or without enzymes, for 30 mins at 37 °C. Cells were then stained with anti-actin, DAPI and Alexa Fluor 594-streptavidin conjugates, and imaged after washing off the free dyes. For Immunofluorescent staining of human lung tissue, paraffin-embedded lung tissue specimens (LCN241) from different healthy human donors were purchased from commercial supplier (US Biomax, Inc.) and used following its recommondations. One antibody (anti-human/mouse CLA antibody, 1:250) and three lectins (AAL, MAL, and SNA, 20 μg/mL) were utilized  for the detection of sLe^X^ epitopes, α1-3-fucosylation, α2-3-linked or α2-6-linked sialylation, respectively. In brief, after the deparaffinizing, rehydrating and immunoblocking the sections, the tissue samples were randomly assigned into four group for this assay and incubated with antibody or lectins in dark on ice for 2 h. The resulted biotin was further stained with Alexa Fluore 647-streptavidin conjugates. Then, the slides were washed and stabilized with mounting medium containing DAPI, before subjected to fluorescence microscopy.

### PNGaseF treatment and western blotting

CHO, CHO-Lec2, and CHO-Lec 8 cells were labeled by Pm2,3ST-M144D, Pd2,6ST, or Hp1,3FT with CMP-Sia*N*Az-biotin or GDP-FucAz-biotin for 30 mins at 37 °C, washed twice with PBS, and lysed on ice in NP-40 lysis buffer. The lysates were then denatured and treated with PNGaseF according to NEB PNGaseF protocols. The western blot was probed with HRP-conjugated anti-biotin IgG.

### Modifying Lec2 cell-surface glycan for HA binding assay

HA of HK68 virus was prepared as previously reported^41^. For HA binding assay, CHO-Lec2 cells were seeded into 96-well flat bottom plate at a density of 2 × 10^4^ cells per well. After 24 h-incubation, cells were treated with recombinant bacterial glycosyltransferase (FTs and STs) and corresponding nucleotide sugars (0.5 mM, GDP-Fuc for FTs and CMP-NeuAc for STs), sequential or one-pot incubation to create new glycoepitopes on the cell-surface as depicted in the figures. After this exogenous glycan modification, the cells were washed three time and incubated with PBS buffer containing 2% BSA and HAs (40 μg/mL), human anti-HA-Fc and anti-Fc-HRP at molar ratio of 4:2:1 for 4 h at 4 °C. Then cells were washed gently with washing buffer (PBS containing 0.05% tween 20) three times, incubated with 1x TMB (Invitrogen) for 20 mins at rt, before quenched with 1 M H_2_SO_4_ and quantified on a plate reader.

### Influenza virus A infectivity assay

WSN, HK68 virus, and the mutant viruses were prepared as previously reported^41^. (Aichi68, Perth09, PR8 and SI06 strains were a gift from Dr. James Paulson at TSRI) Host MDCK-cells were seeded into 96-well flat bottom plate at the density of 2 × 10^4^ cells per well. When cell grew to about 80% confluency, MDCK cells were subjected to Pd2,6ST-catalyzed α2,6sialylation, Hp1,3FT-assisted α1-3-fucosylation or not. The influenza A viruses then were diluted in media and incubated with MDCK cells. At 2 h post-infection, cells were washed three times with PBS followed by the addition of fresh medium containing trypsin. After 48 h-incubation, cell viability was quantified using MTS (G3582) method as recommended by the commercial supplier. For cell viability quantification, the samples with microbial containmination were excluded from the statistics.

### Reporting Summary

Further information on experimental design is available in the Nature Research Reporting Summary linked to this article.

## Supplementary information

Supplementary Information

Reporting Summary

Source Data File

## Data Availability

The binding of sLeX with HK68-HA and HK68-MTA-HA was modeled based on aligning the corresponding HA apo structures to the crystal structure of A/canine/Colorado/17864/06 (H3 subtype) HA in complex with sLeX (by PyMOL^57^). Structure information resources  were HK68-HA (PDB 4FNK)^44^, HK68-MTA-HA (PDB 5VTX)^41^, and canine HA^45^. Alignment was performed using the receptor-binding subdomain (residues 117 to 265 of HA1)^46^. The raw data underlying Figs. 1b–i, 2d–g, 4c, 5 and 6a–i, as well as Supplementary Figs. 1A, 2, 3B-E, 4, 9, 10, 12, and 13 are available in the source data file. Other raw data that support the findings of this study are available from the authors on reasonable request.
