# Supplementary material for: Bacterial glycosyltransferase-mediated cell-surface chemoenzymatic glycan modification
Source: Nat Commun. 2019 Apr 17;10:1799. doi: 10.1038/s41467-019-09608-w (PMC6470217; doi:10.1038/s41467-019-09608-w)
Supplement: Supplementary file 1 — Supplementary Information [file 41467_2019_9608_MOESM1_ESM.pdf]

Supplementary Information

Bacterial Glycosyltransferase-mediated Cell-surface Chemoenzymatic Glycan Modification

Hong, *et al*

Contents:

Supplementary Figures 2

Supplementary Figure 1 2

Supplementary Figure 2 2

Supplementary Figure 3 3

Supplementary Figure 4 4

Supplementary Figure 5 4

Supplementary Figure 6 5

Supplementary Figure 7 5

Supplementary Figure 8 6

Supplementary Figure 9 7

Supplementary Figure 10 7

Supplementary Figure 11 8

Supplementary Figure 12 9

Supplementary Figure 13 9

Supplementary Figure 14. 10

Supplementary Figure 15. 11

Supplementary Figure 16. 12

Supplementary Figure 17. 13

Supplementary Figure 18. 14

Supplementary Figure 19. 15

Supplementary Figure 20. 16

Supplementary Figure 21. 17

Supplementary Figure 22. 18

Supplementary Figure 23. 19

Supplementary Figure 24. 20

Supplementary Methods 21

Supplementary References 22

Supplementary Figures

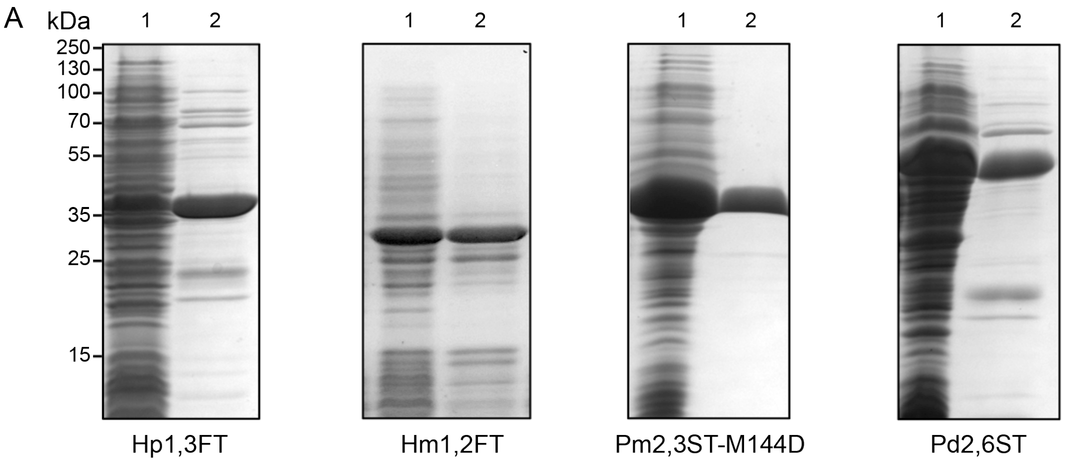

B

Enzyme	Antibiotics (µg/mL)	Induction (IPTG)	Storage	Yield (/L)
Hp1,3FT	kanamycin (25)	4 h, 25 °C	50 mM Tris-HCl<br>pH 8.0<br>100 mM NaCl<br>1 mM DTT<br>5% glycerol	9 mg
Hm1,2FT	kanamycin (25)	16 h, 16 °C		1 mg*
Pm2,3ST-M144D	ampicillin (100)	16 h, 20 °C		15 mg
Pd2,6ST	kanamycin (25)	3 h, 37 °C		15 mg

Supplementary Figure 1. Production conditions of recombinant bacteria glycosyltransferases in *E. coli* expression system. (A) SDS-PAGE analysis of recombinant bacteria glycosyltransferases produced in an *E. coli* expression system. Lane 1, *E. coli* lysate before purification; Lane 2, Purified enzymes. (B) Expression and storage conditions for four glycosyltransferases. * The low yield of Hm1,2FT was due to the formation of inclusion bodies. Source data for the figure A are provided as a Source Data file.

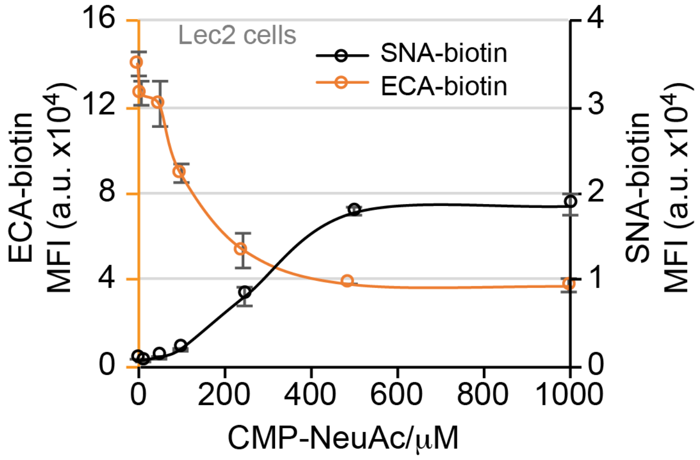

Supplementary Figure 2. Flow cytometry analysis of the Pd2,6ST-assisted incorporation of CMP-NeuAc onto MDCK. MDCK cells were incubated in HBSS buffer containing 3 mM HEPES, 20 mM Mg2+, different concentrations of CMP-NeuAc (0, 10, 50, 100, 250, 500 or 1000 µM), and Pd2,6ST, for 30 mins. Then, the chemoenzymatically incorporated cell-surface Sia was stained with lectins and quantified with flow cytometry. SNA-biotin is specific for onto MDCK cell-surface. MDCK cells were incubated in HBSS buffer containing 3 mM HEPES, 20 mM Mg2+, different cacetylgalactosamine (GalNAc) and lactose. Error bars represent the standard deviation of three biological replicates. Source data are provided as a Source Data file.

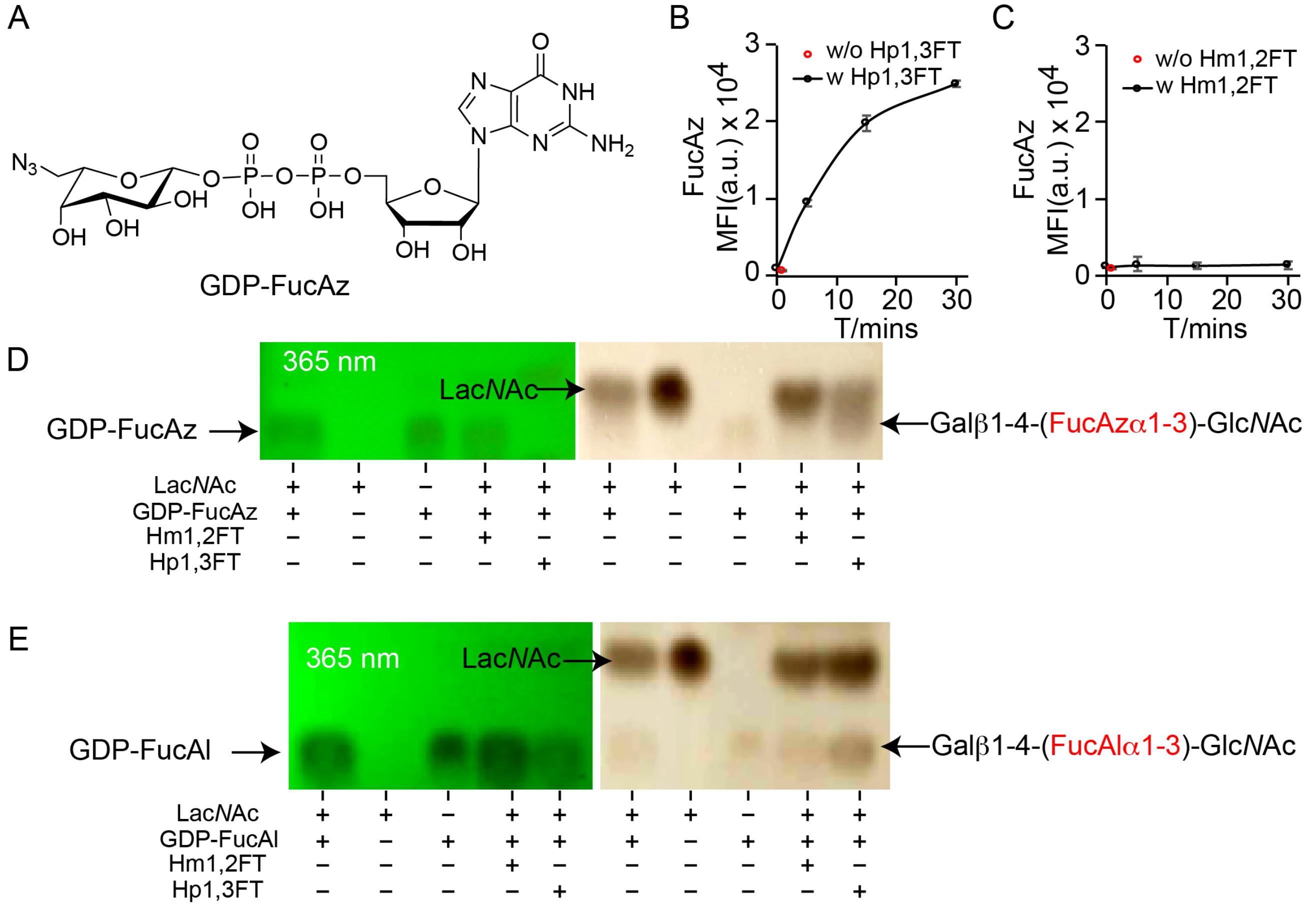

Supplementary Figure 3. Evaluation of the substrate tolerance of bacterial fucosyltransferases. (A)For FTs, GDP-FucAz unnatural sugar bearing the azide group was tested. (B and C) Flow cytometry analysis of FT-assisted chemoenzymatic labeling of Lec2 cell. Lec2 cells were incubated with a fucosyltransferase (Hp1,3FT or Hm1,2FT) and GDP-FucAz. Following the enzymatic treatment, the modified cells were reacted with an alkynyl biotin via the ligand (BTTPS)-assisted copper-catalyzed azide-alkyne [3+2] cycloaddition reaction (CuAAC), and probed with Alexa Fluor 488-streptavidin conjugate. Hp1,3FT-treated cells exhibited significant labeling of time-dependented (B), and no fluorescent signals were detectable for the Hm1,2FT treated cells (C). Error bars represent the standard deviation of three biological replicates. (D and E) TLC analysis of the donor substrate scope of FTs. Two unnatural GDP-Fuc analogs were tested for *in vitro* fucosylation of Lac*N*Ac catalyzed by Hm1,2FT and Hp1,3FT, including GDP-FucAz (D) and GDP-FucAl (E). For TLC analysis, isopropanol: H_2O: NH_4OH (8:3:2) was used as the development solvent, the nucleotide sugar was visualized under a 365nm ultraviolet lamp, and Lac*N*Ac and the products were visualized by staining with 10% sulfuric acid in ethanol. Source data for the figure B-E are provided as a Source Data file.

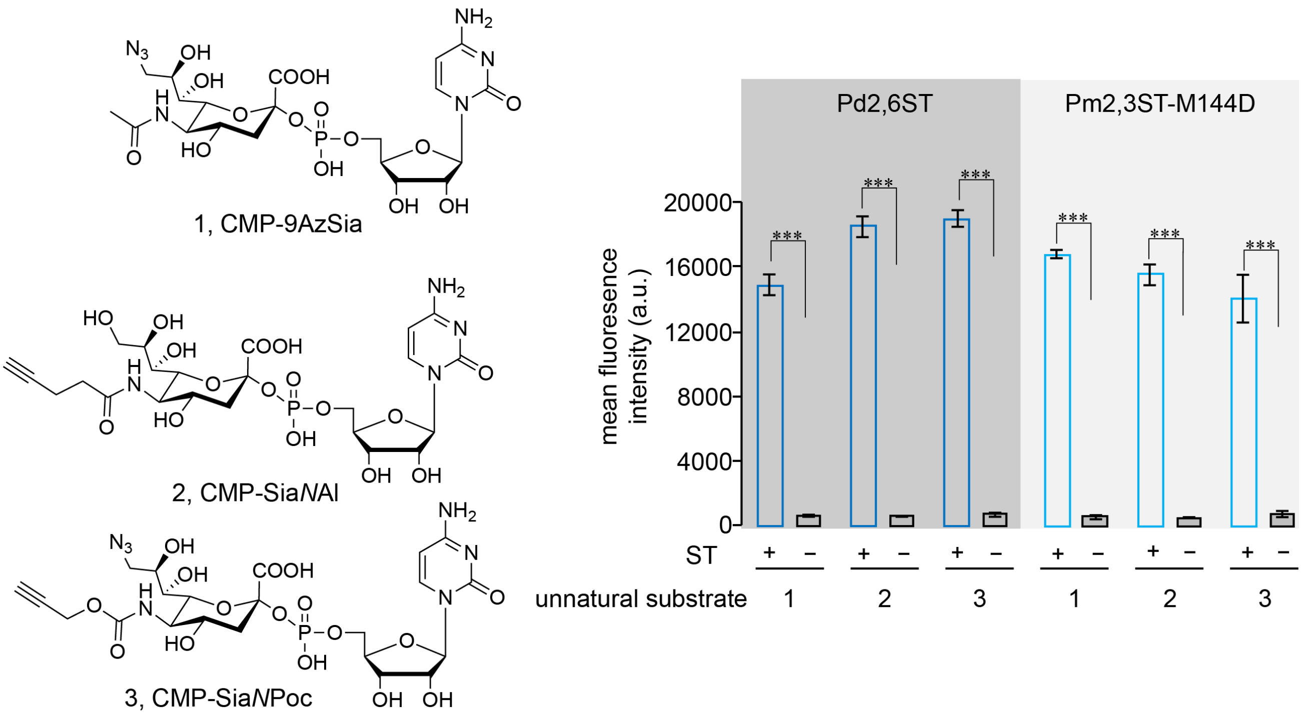

Supplementary Figure 4. Evaluation of the donor substrate scope of Pm2,3ST-M144D and Pd2,6ST. Three types of unnatural CMP-Sia (CMP-Sia*N*Al, CMP-Sia*N*Poc and CMP-9AzSia) were tested in STs-catalyzed modification of Lec2 cells. The chemoenzymatically incorporated unnatural chemical reporters (alkyl or azido groups) were derived into biotin using ligand-assisted CuAAC reaction with complementary functional group (azido or alkyl) modified biotin. Then the biotins were stained with Alexa Fluor 647-streptavidin conjugate and quantified with flow cytometry. Error bars represent the standard deviation of three biological replicates. *** indicated Student's T-test P<0.001. Source data are provided as a Source Data file.

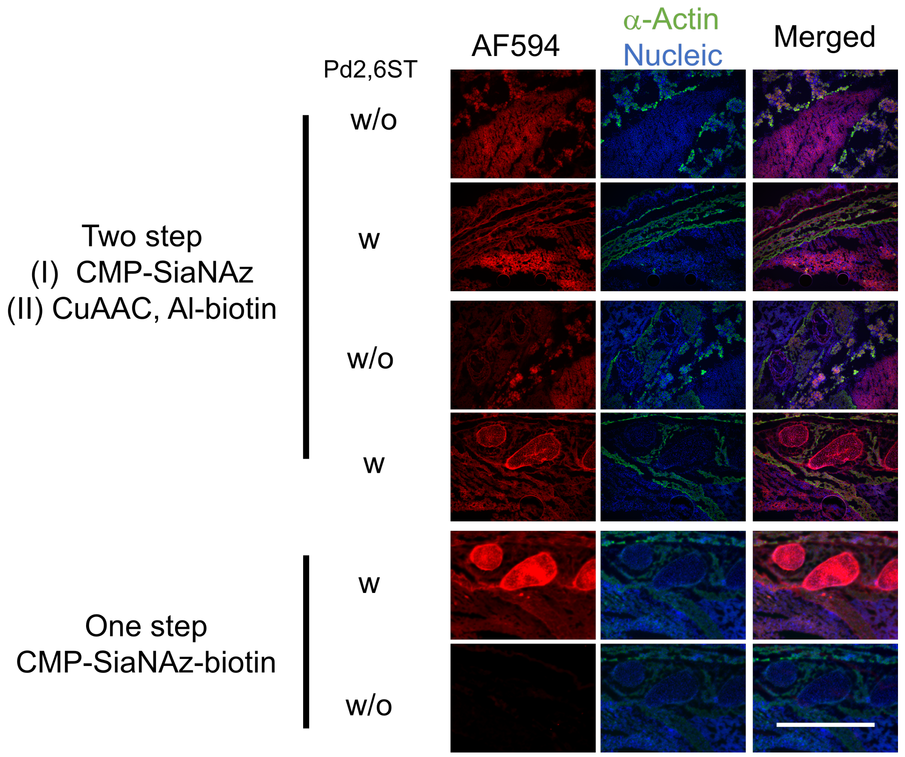

Supplementary Figure 5. Comparison of the One-step tissue glycan labeling with traditional two-step strategy. The One-step tissue glycan labeling enabled by Pd2,6ST-mediated incorporation of unnatural sugars conjugated to an affinity tag (biotin), was directly compared with traditional two-step strategy using azide-bearing unnatural sugars followed by CuAAC-conjugation of biotin. The embryonic frozen sections from E16 mouse were incubated with Pd2,6ST or without ST, and CMP-Sia*N*Az-biotin or CMP-Sia*N*Az. The biotin was further probed by Alexa Fluor 594-streptavidin conjugate staining. The resulting fluorescence (red) of different parts of the embryo was directly imaged using microscopy, including abdominal region and anterior chest. The cells of frozen sections were stained with anti-actin (green) and DAPI (blue, nucleus). Scale bar, 1 mm.

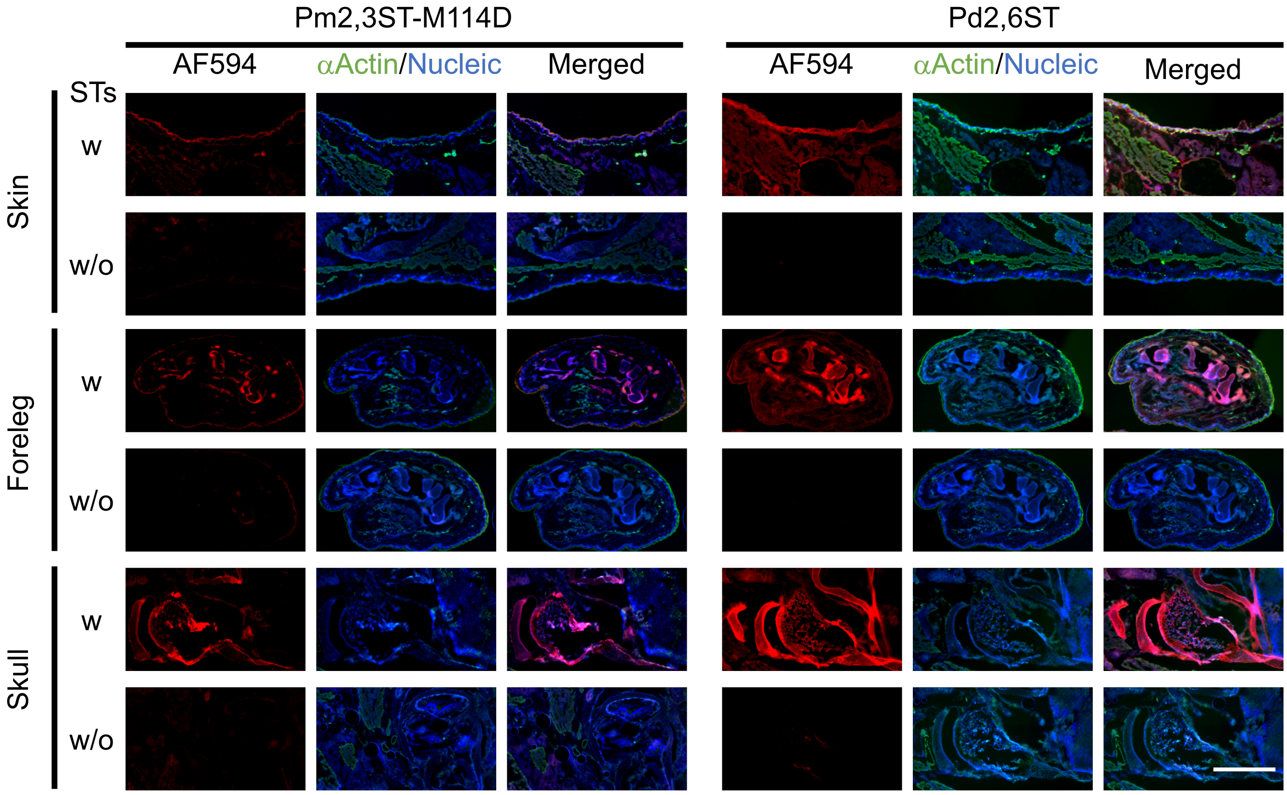

Supplementary Figure 6. One-step chemo-enzymatic labeling of glycans on tissue specimens of mouse embryo. The embryonic frozen sections from E16 mouse were incubated with STs or without STs, and CMP-Sia*N*Az-Biotin, followed by Alexa Fluor 594-streptavidin conjugate staining. The resulted fluorescence (red) of different parts of embryo was directly imaged using microscopy, including skin regions, forelimb and skull. The cells of frozen sections were stained with anti-actin (green) and DAPI (blue, nucleus). Scale bar, 1 mm.

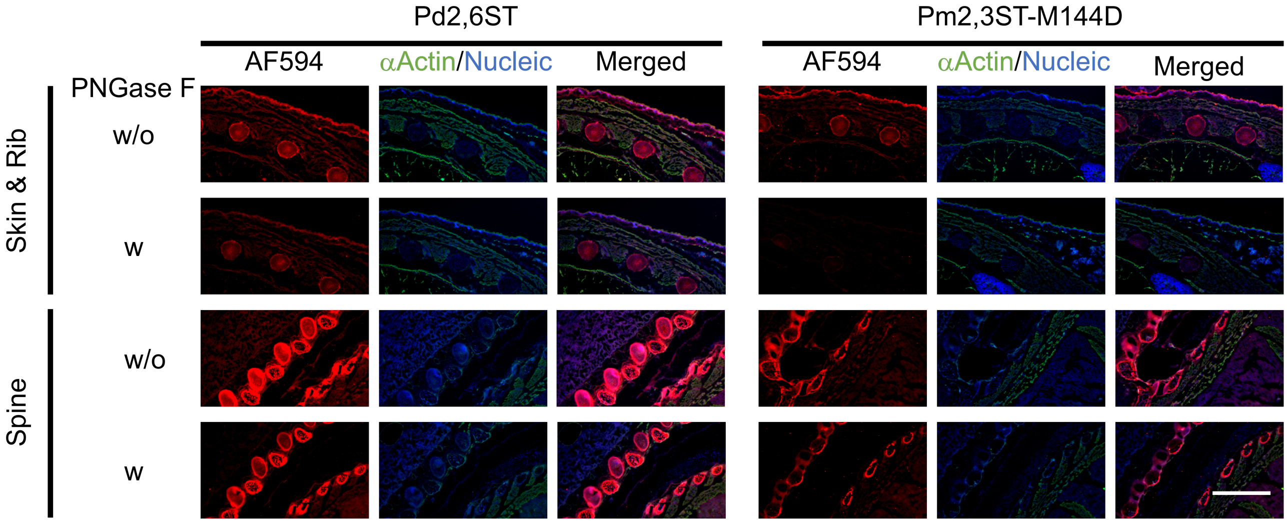

Supplementary Figure 7. One-step labeling of glycans on tissue specimens after PNGase F-releasing of *N*-glycans. The embryonic frozen sections from E16 mouse were fixed and incubated with or without PNGase F. Then, tissues were labeled with STs and CMP-Sia*N*Az-Biotin, followed by Alexa Fluor 594-streptavidin conjugate staining. The resulted fluorescence (red) of different parts of embryo was directly imaged using microscopy, including skin, rib and spine regions. The cells of frozen sections were stained with anti-actin (green) and DAPI (blue, nucleus). Scale bar, 1 mm.

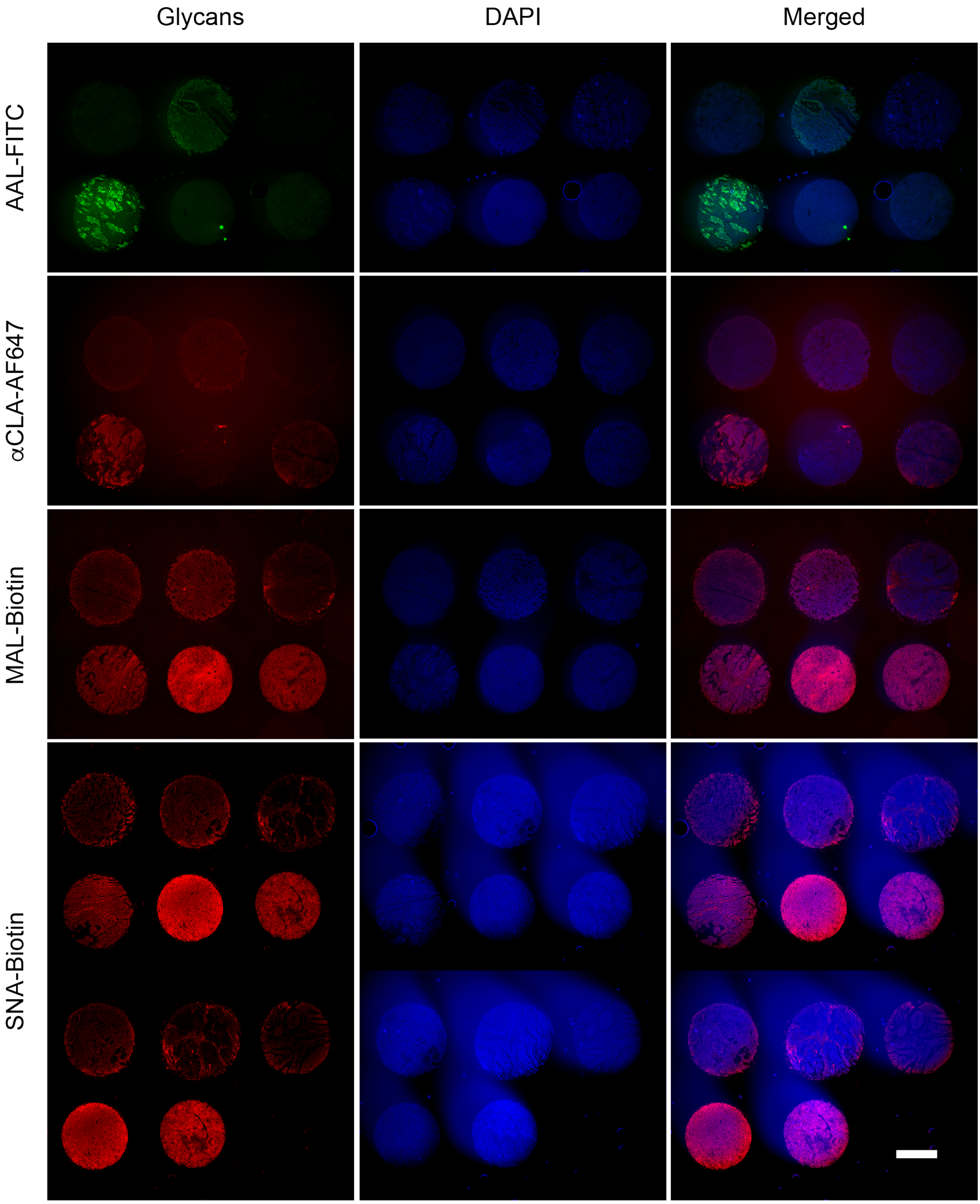

Supplementary Figure 8. Profiling glycoforms of lung tissues obtained from healthy human donors. Lung tissue slides were stained with FITC-AAL, AF647-anti-CLA, Biotin-MAA or Biotin-SNA conjugates to detect α1-3-fucosylation, sLe^X epitopes, α2-3-linked or α2-6-linked sialylation, respectively. Formalin-fixed paraffin embedded lung sections from different health peoples were purchased from commercialized supplier. After washing off the paraffin, slides were incubated with lectins overnight at 4 ^{o}C. AAL-FITC binds preferentially to fucose that is α1-6-linked to *N*-acetylglucosamine or α1-3-linked to *N*-acetyllactosamine related structures; anti-CLA Alexa Flour 647 (αCLA-AF647) recognizes sialofucosylated glycans and sLe^X; MAL lectin binds preferentially to α2,3-linked sialylated glycans, and SNA lectin binds preferentially to α2,6-linked sialylated glycans. The biotin conjugated on lectins were further stained with Alexa Fluor 594-streptavidin conjugate. Tissue cell nucleus was stained with DAPI. Then, samples were imaged by fluorescent microscopy. Scale bar, 1 mm.

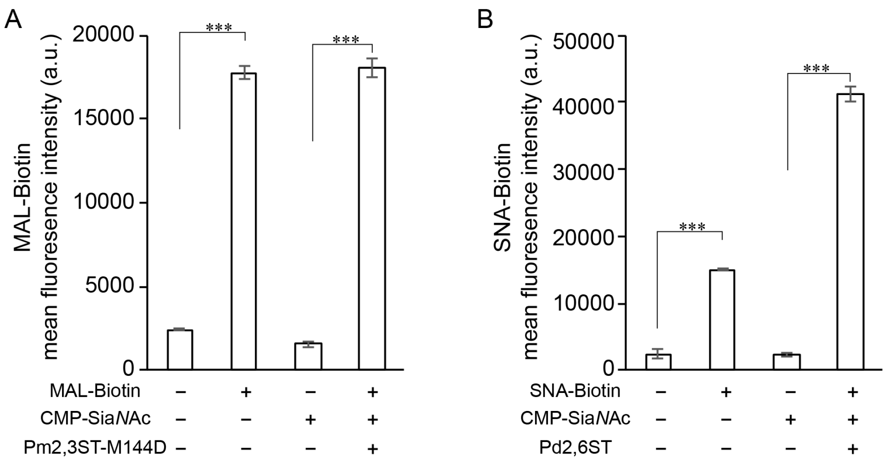

Supplementary Figure 9. Lectin staining analysis of newly formed glycan epitopes on the MDCK cell-surface. After chemoenzymatic glycan modification, the cells were stained with lectin (MAL or SNA) to probe newly formed glycan epitopes. (A) MDCK cell surface α2-3-linked Sia was stained with MAL-biotin (10 μg/mL). (B) MDCK cell surface α2-6-linked Sia was stained with SNA-biotin (10 μg/mL). Cell-surface biotinylated glycans, then, were probed by Alexa Fluore 647-streptavidin conjugate, and quantified by flow cytometry. The error bars represent the standard deviation of three biological replicates. *** indicated Student's T-test P<0.001. Source data are provided as a Source Data file.

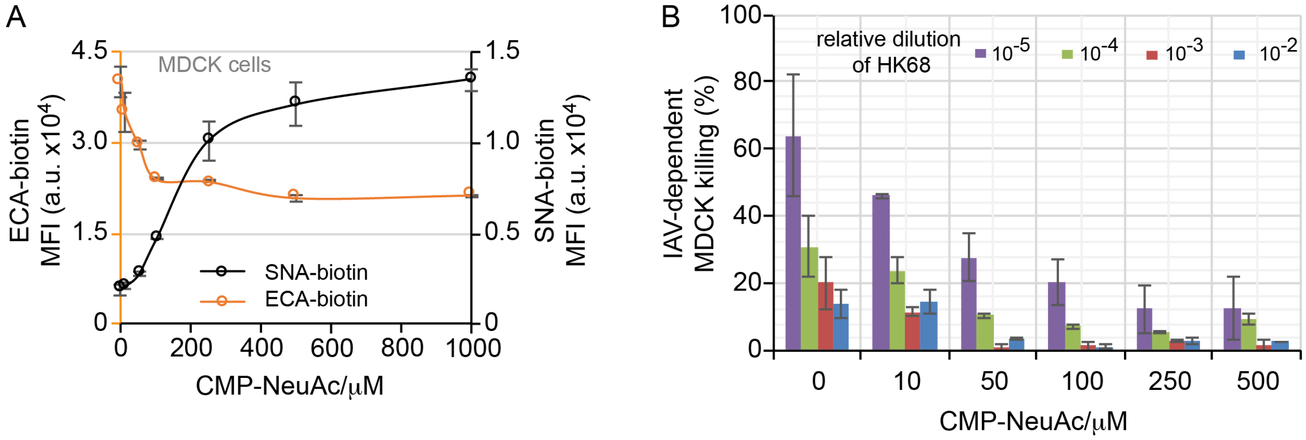

Supplementary Figure 10. Pd2,6ST-assisted glycocalyx modification of live MDCK cells. (A) Pd2,6ST-catalyzed dose-dependent incorporation of natural CMP-NeuAc onto live MDCK cells was monitered by lectin staining and flow cytometry. The error bar presented the standard deviation of three biological replicates. SNA-biotin is specific for αNA-biotin is specific for standar*Erythrina Cristagalli* lectin (ECA-biotin) is specific for galactose (Gal), *N*-acetylgalactosamine (Gal*N*Ac) and lactose. Cells were incubated in HBSS buffer containing 3 mM HEPES, 20 mM Mg^{2+}, different concentrations of CMP-NeuAc (0, 10, 50, 100, 250, 500 or 1000 μ), and Pd2,6ST, for 30 mins. Then, the chemoenzymatically incorporated Sias were stained with lectins and quantified with flow cytometry. (B) Viability of Sia-edited MDCK cells with different concentration of CMP-NeuAc or control cells, upon infection by wild-type HK68 at different virus dlutions, including 10^{-2}, 10^{-3}, 10^{-4} and 10^{-5} virus dilution. The cell viablility was measured after 3-day virus-host coincubation. The error bars represent the standard deviation of six biological replicates. Source data are provided as a Source Data file.

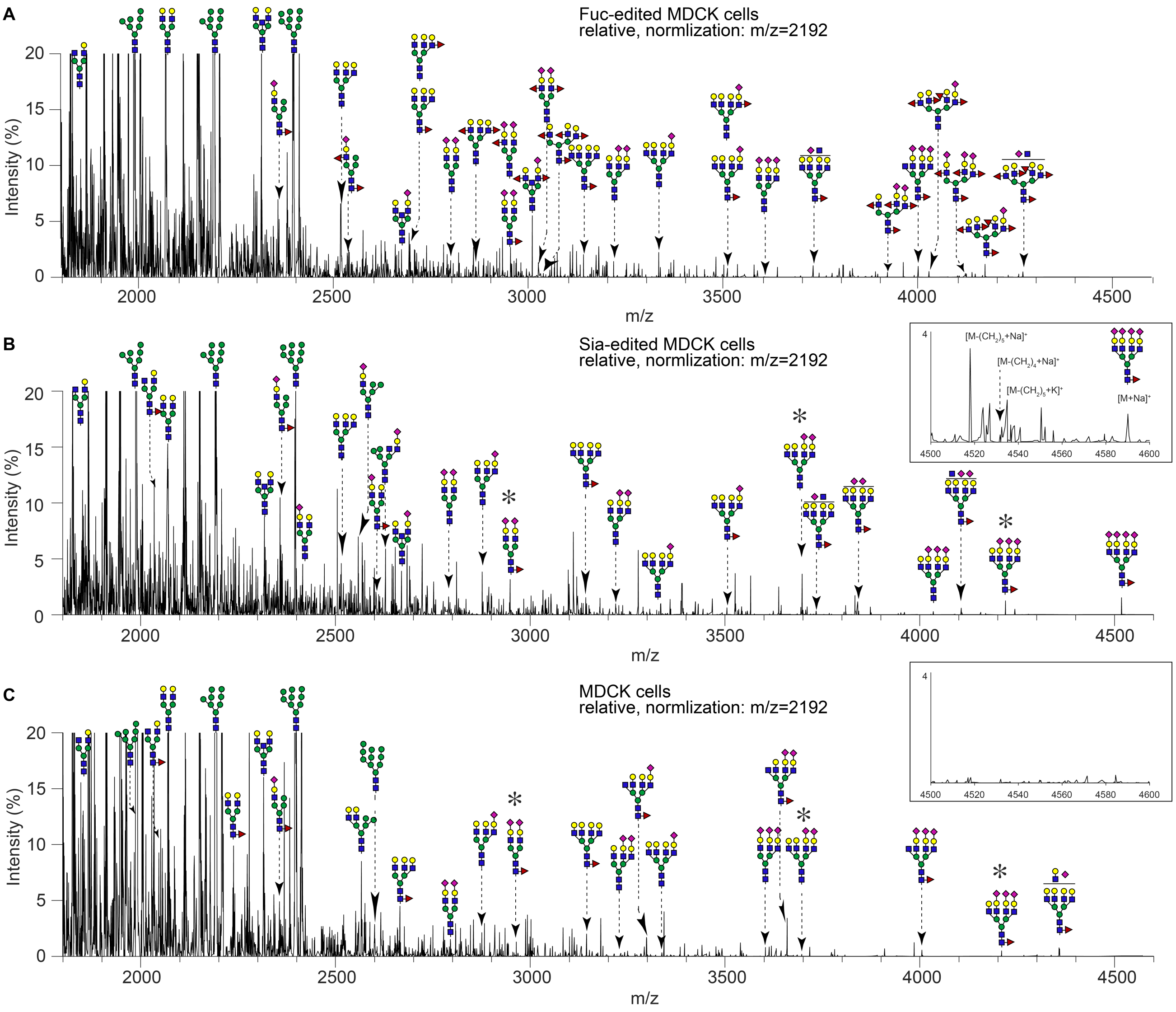

Supplementary Figure 11. Glycomics analysis of MDCK cell-surface *N*-linked glycan. The permethylated *N*-linked glycans from Hp1,3FT-edited, Pd2,6ST-edited or untreated MDCK cells were analyzed by the matrix assisted laser desorption/ionization time-of-flight (MALDI-TOF) mass spectrometry (MS). MDCK cells were treated with Hp1,3FT/GDP-Fuc (A), Pd2,6ST/CMP-NeuAc (B) or not (C) at 37 °C for 30 mintures. Next, the cells were lysated, and *N*-linked glycans of glycoproteins from each sample were released form peptides with PNGase F after the typsin-digestion. Then, the glycans were isolated, permethylated, and analyzed by MALDI-TOF MS. All molecular ions are present in sodiated form ($[M+Na]^+$), and the putative structures present here are based on composition and biosynthetic knowledge. Structures that show sugars outside of a bracket have not been unequivocally defined. The intensity of the m/z=2192 peak in each spectrum was normalized to 100%.

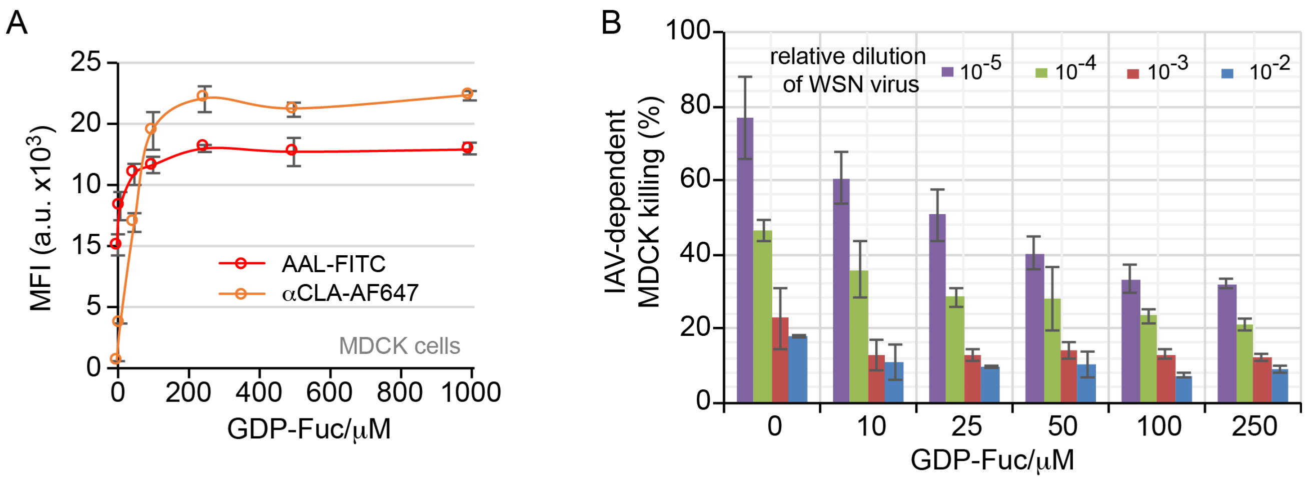

Supplementary Figure 12. Hp1,3FT-assisted glycocalyx modification of host MDCK cells. (A) Hp1,3FT-catalyzed dose-dependent incorporation of natural GDP-Fuc onto live MDCK cells was monitered by lectin staining (AAL-FITC) and antibody staining (αCLA-AF647). The relative fluorensent intensity was quantified by flow cytometry. The error bar presented the standard deviation of three biological replicates. AAL-FITC is specific for α1,3-linked Fuc. Cells were incubated in HBSS buffer containing 20 mM HEPES, 3 mM Mg^{2+}, different concentrations of GDP-Fuc (0, 10, 25, 50, 100, 250 or 500 μM), and Hp1,3FT, for 30 mins. Then, the chemoenzymatically incorporated Fuc were stained with lectins or antibody, and quantified with flow cytometry. (B) Viability of Fuc-edited MDCK cells with different concentration of GDP-Fuc or control cells, upon infection by WSN at different virus dilutions of 10^{-2}, 10^{-3}, 10^{-4} and 10^{-5}. The cell viablility was measured after 2-day virus-host coincubation. The error bars represent the standard deviation of six biological replicates. Source data are provided as a Source Data file.

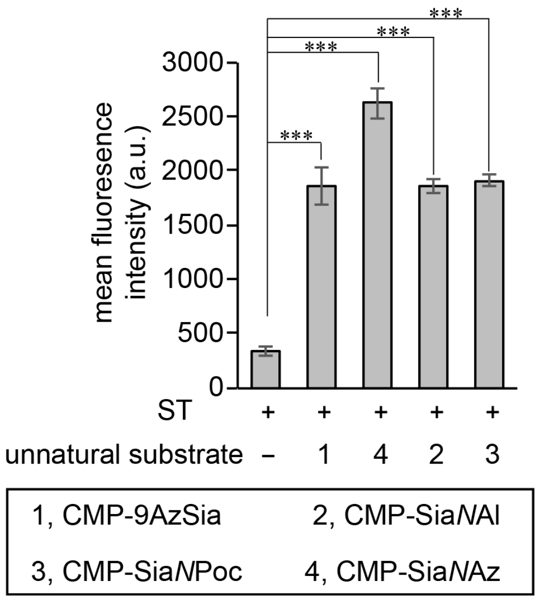

Supplementary Figure 13. Flow cytometry analysis of the Pd2,6ST-assisted incorporation of unnatural CMP-Sias. MDCK cells were incubated in HBSS buffer containing 3 mM HEPES, 20 mM Mg^{2+}, 200 μM CMP-Sias (CMP-Sia*N*Ac, CMP-Sia*N*Az, CMP-Sia*N*Al, CMP-Sia*N*Poc or CMP-9AzSia), and Pd2,6ST or without enzyme, for 30 mins. Then, the chemoenzymatically incorporated unnatural chemical reporters (alkyl and azido groups) on MDCK cell-surface were derived into biotin using ligand-assisted CuAAC reaction with complementary functional group modified biotin. Then the biotins were stained with Alexa Fluor 647-streptavidin conjugate and quantified with flow cytometry. Error bars represent the standard deviation of three biological replicates. *** indicated Student's T-test P<0.001. Source data are provided as a Source Data file.

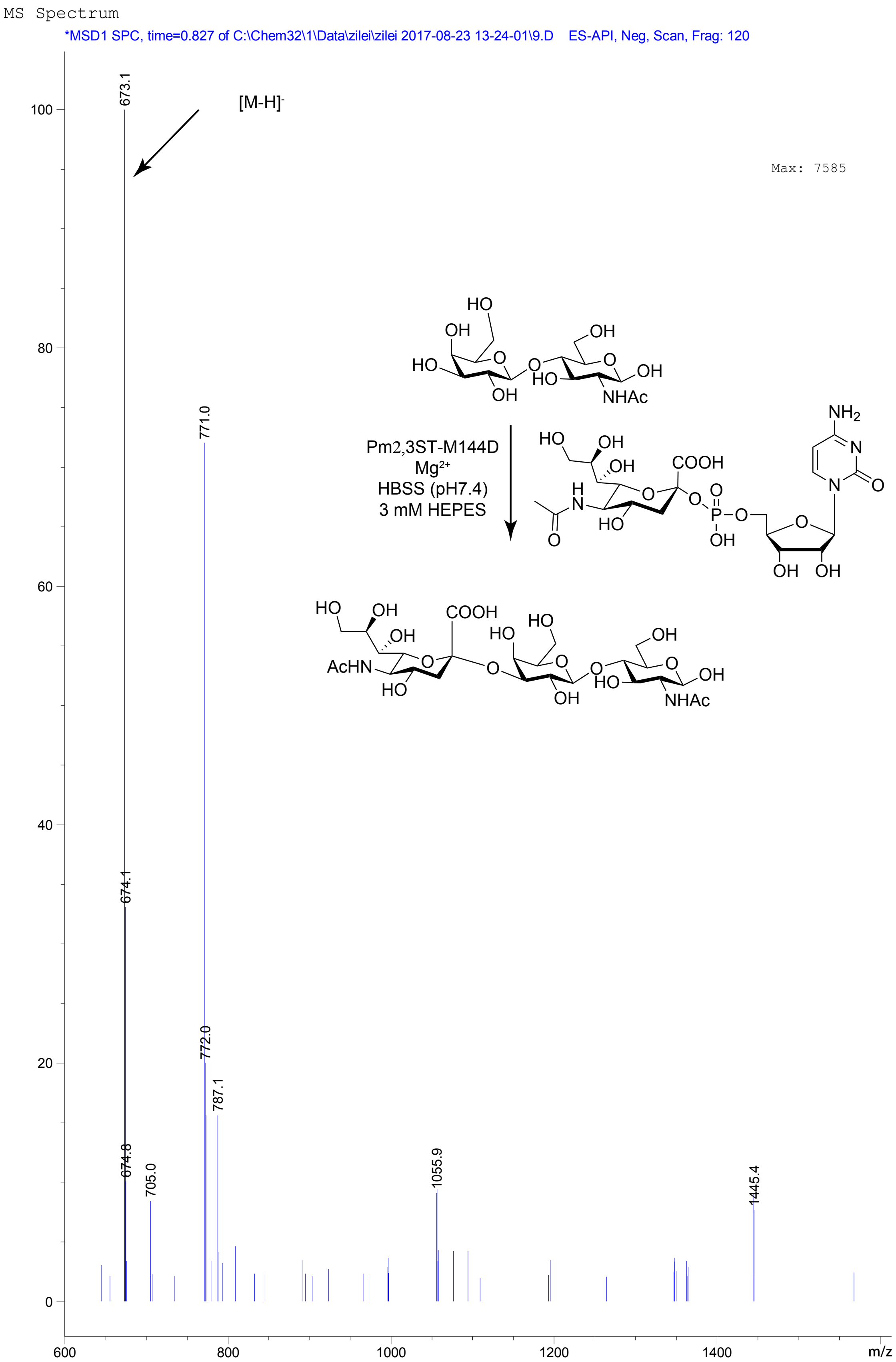

Supplementary Figure 14. Mass spectral characterization of Neu5Acα2-3-Galβ1-4-GlcNAc via LC/MS.

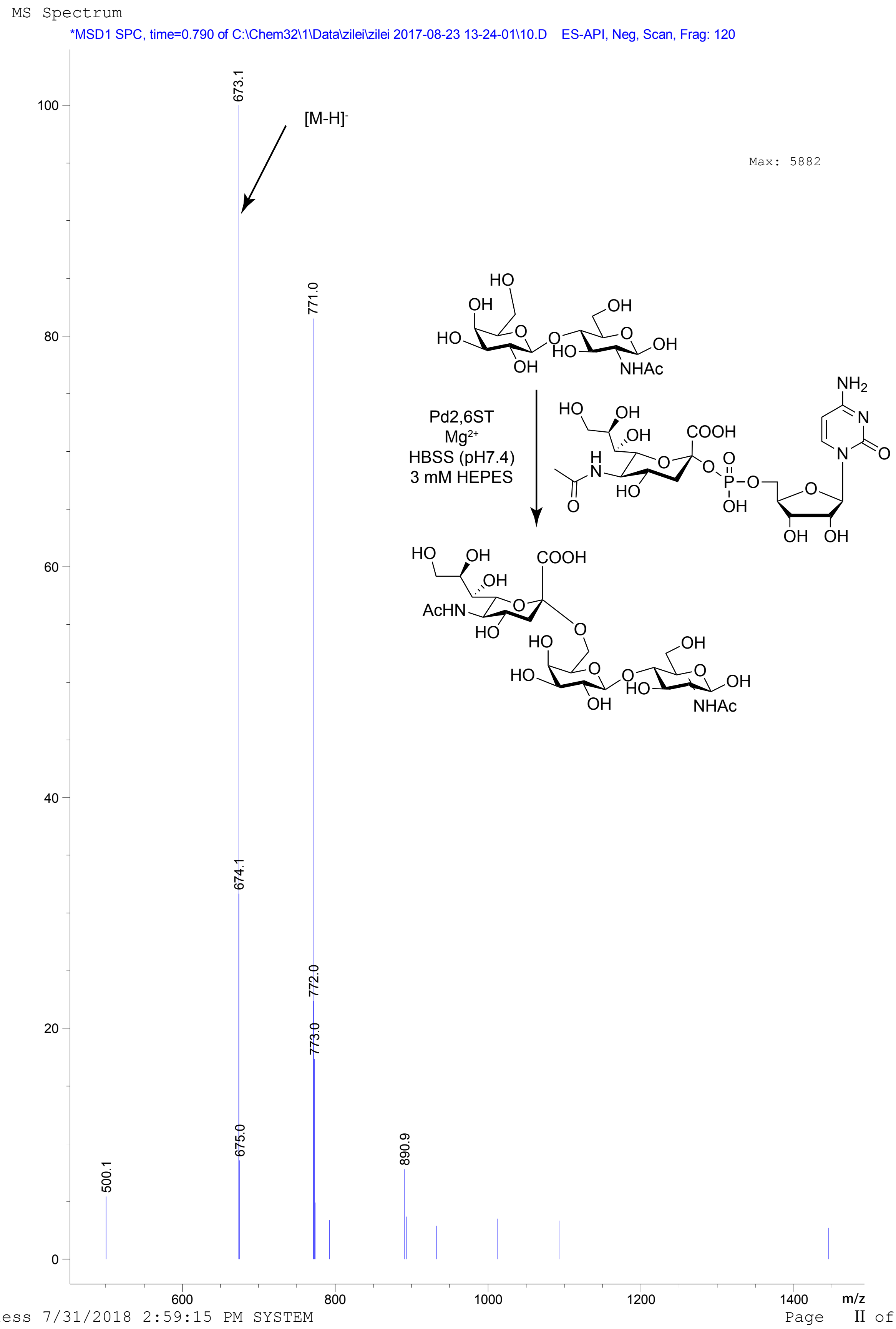

Supplementary Figure 15. Mass spectral characterization of Neu5Acα2-6-Galβ1-4-GlcNAc via LC/MS.

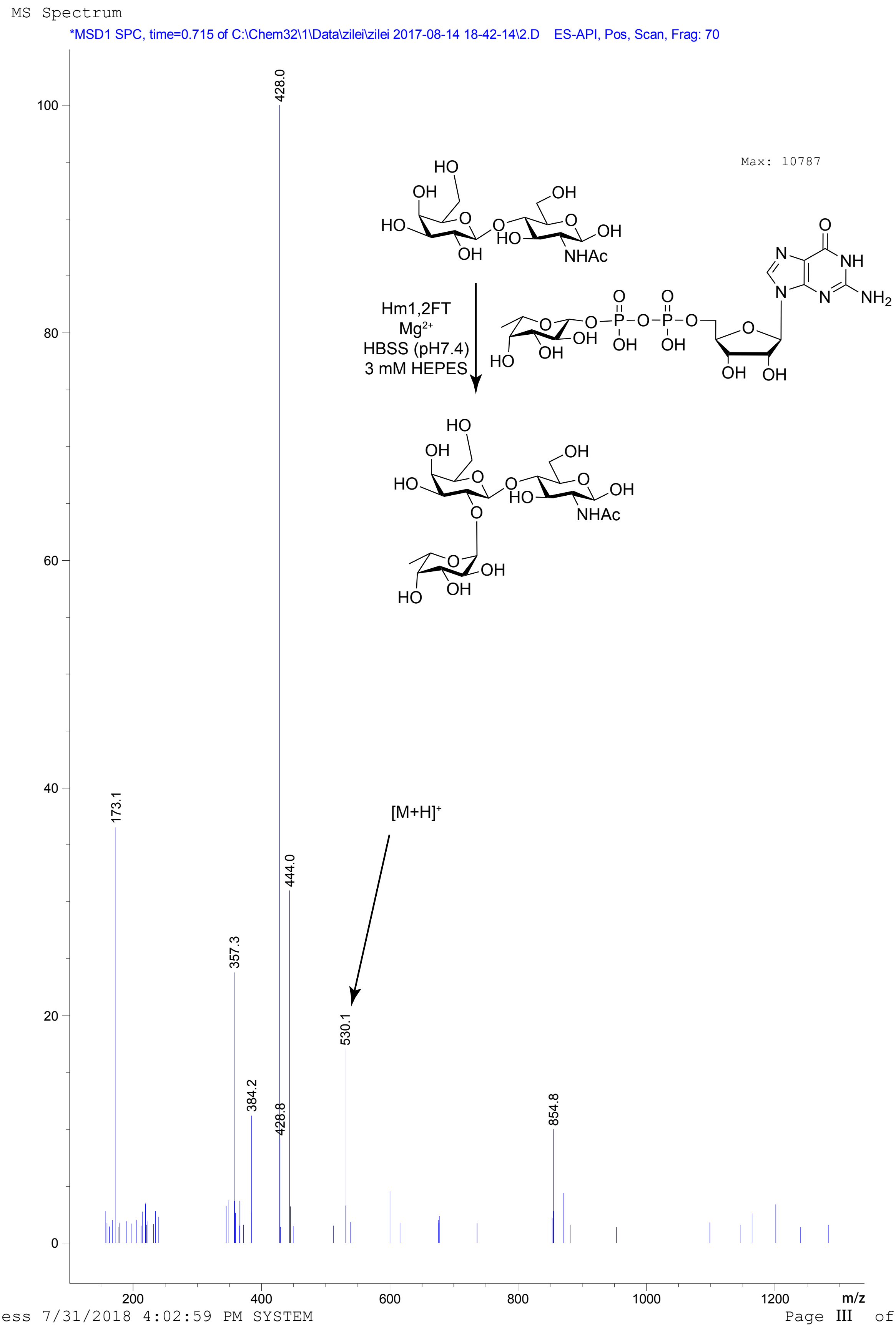

Supplementary Figure 16. Mass spectral characterization of Fucα1-2-Galβ1-4-GlcNAc via LC/MS.

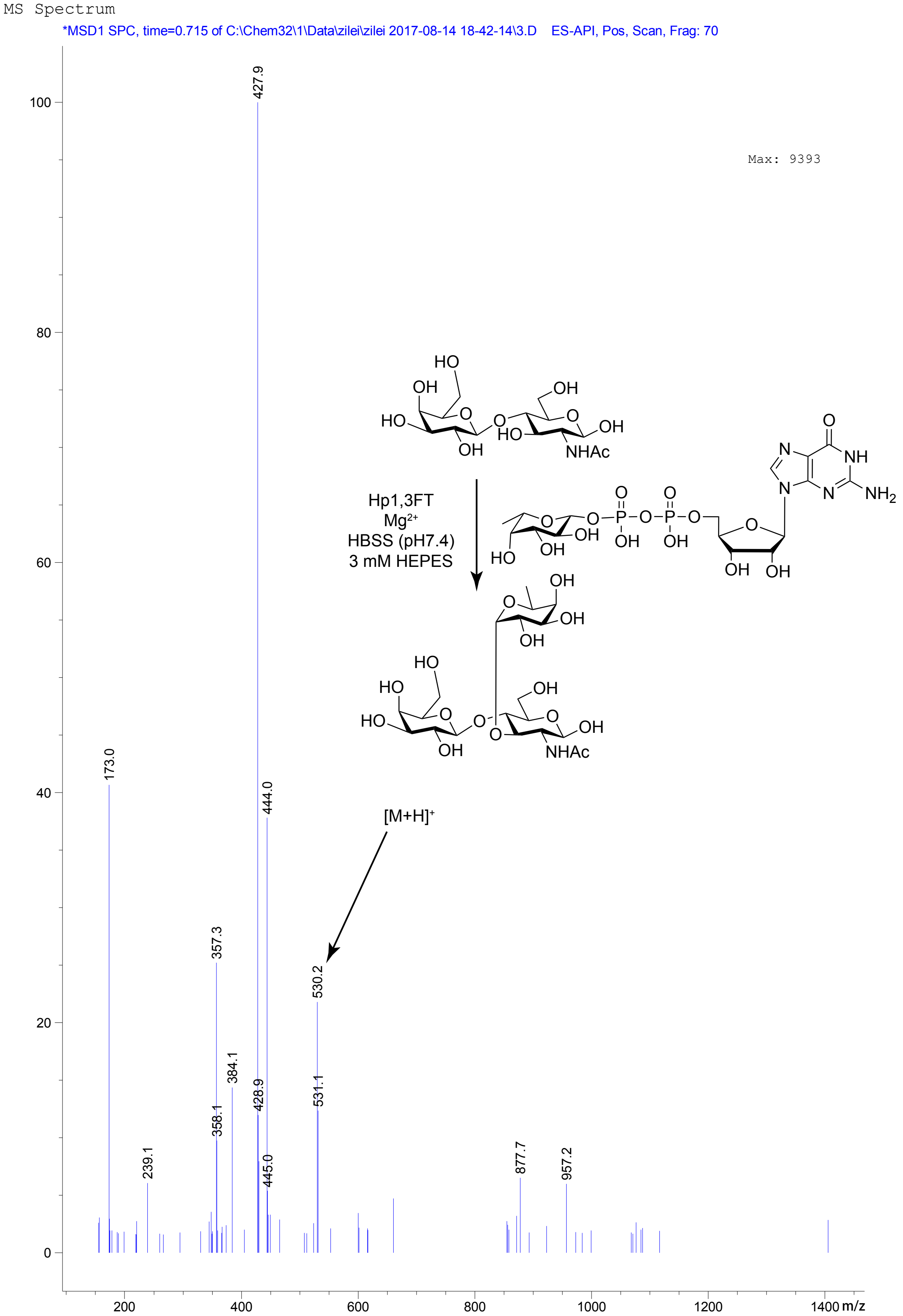

Supplementary Figure 17. Mass spectral characterization of Le^{X} (Galβ1-4(Fucα1-3)-GlcNAc via LC/MS.

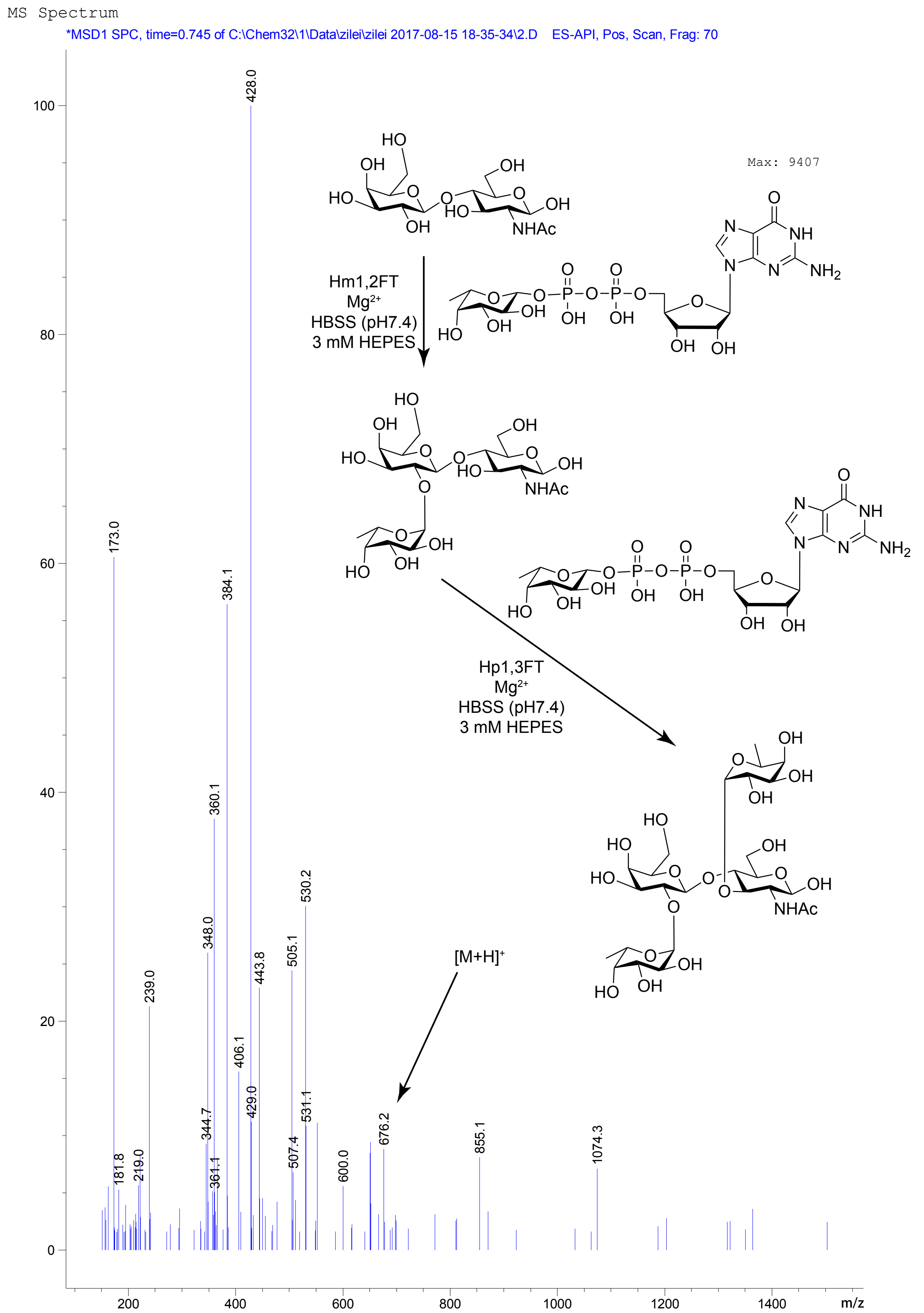

Supplementary Figure 18. Mass spectral characterization of Fucα1-2-Galβ1-4(Fucα1-3)-GlcNAc (Le^{Y}) via LC/MS.

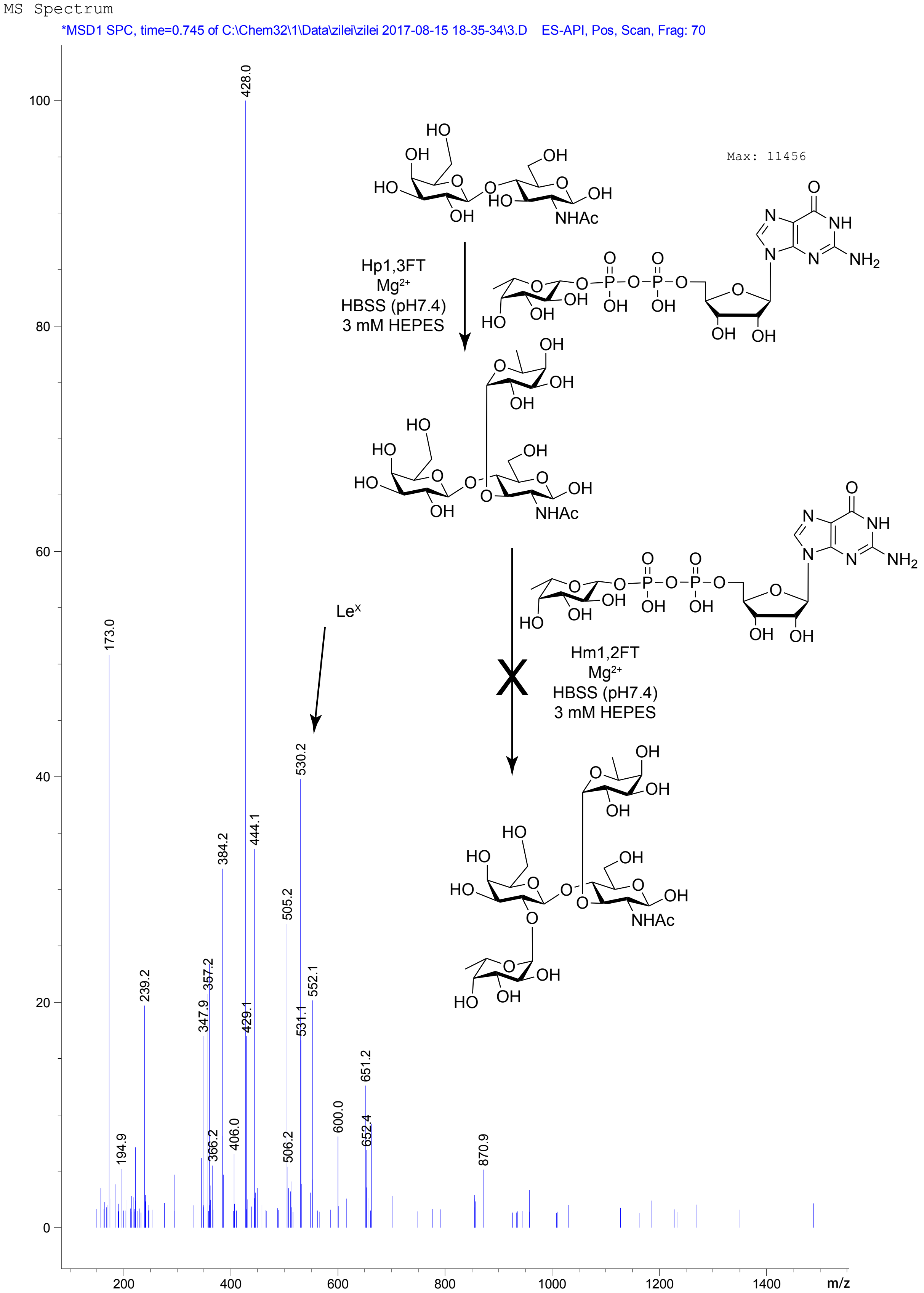

Supplementary Figure 19. LC/MS monitering the chemoenzymatic synthesis of Le^{Y}.

MS Spectrum

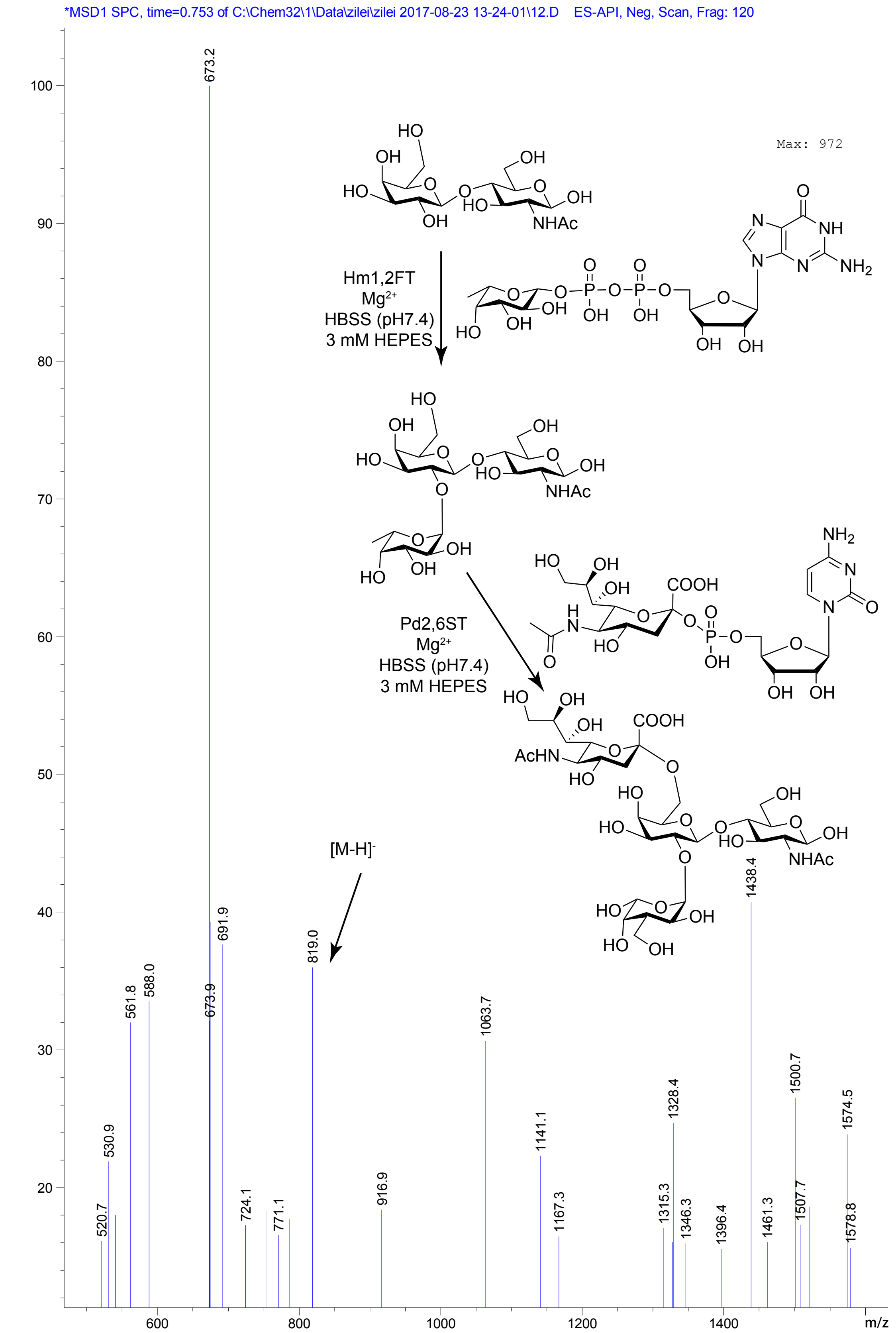

Supplementary Figure 20. Mass spectral characterization of NeuAcα2-6(Fucα1-2)-Galβ1-4-GlcNAc via LC/MS.

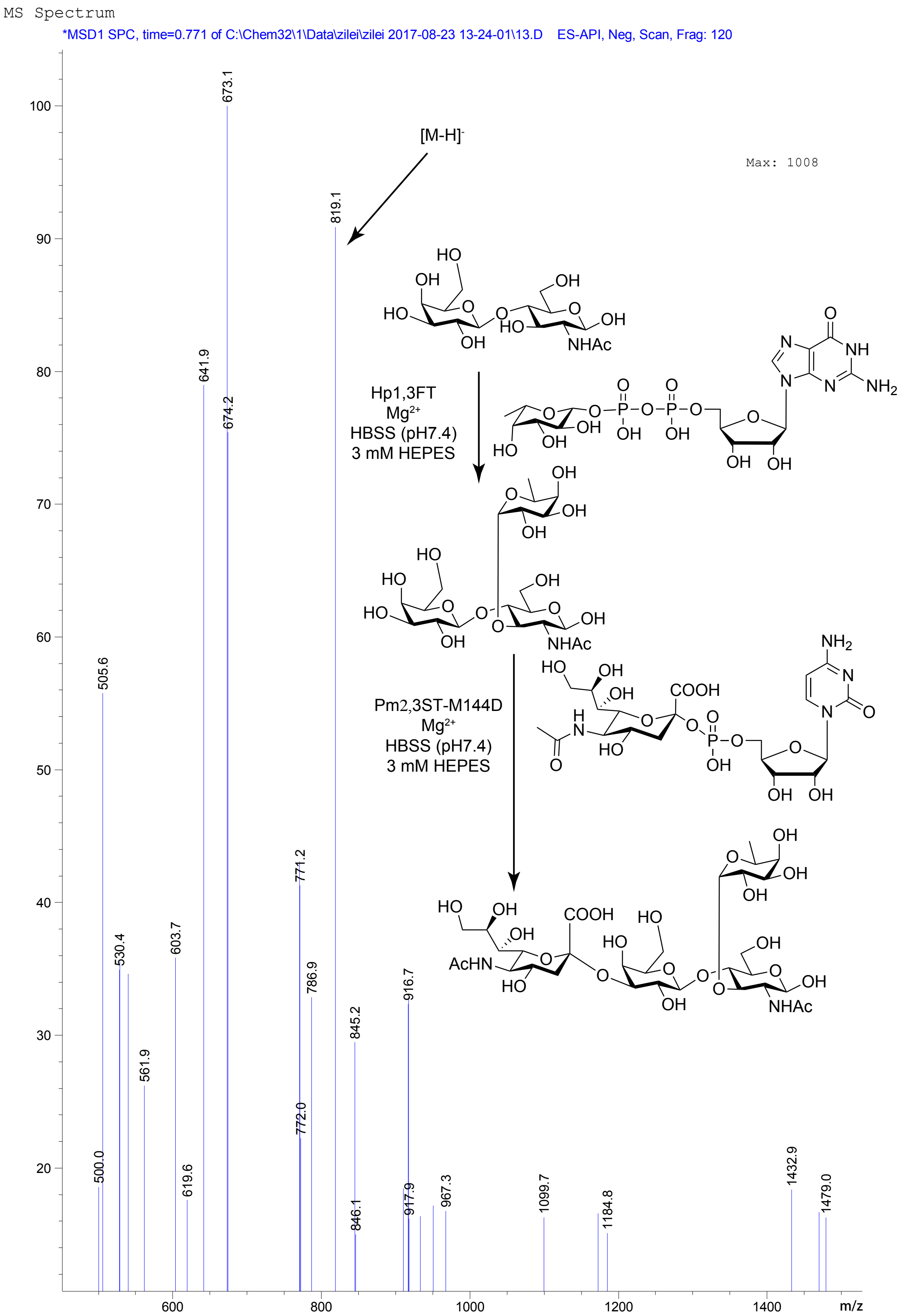

Supplementary Figure 21. Mass spectral characterization of sLe^{X} NeuAcα2-3-Galβ1-4(Fucα1-3)-GlcNAc via LC/MS.

Print of window 80: MS Spectrum

MS Spectrum

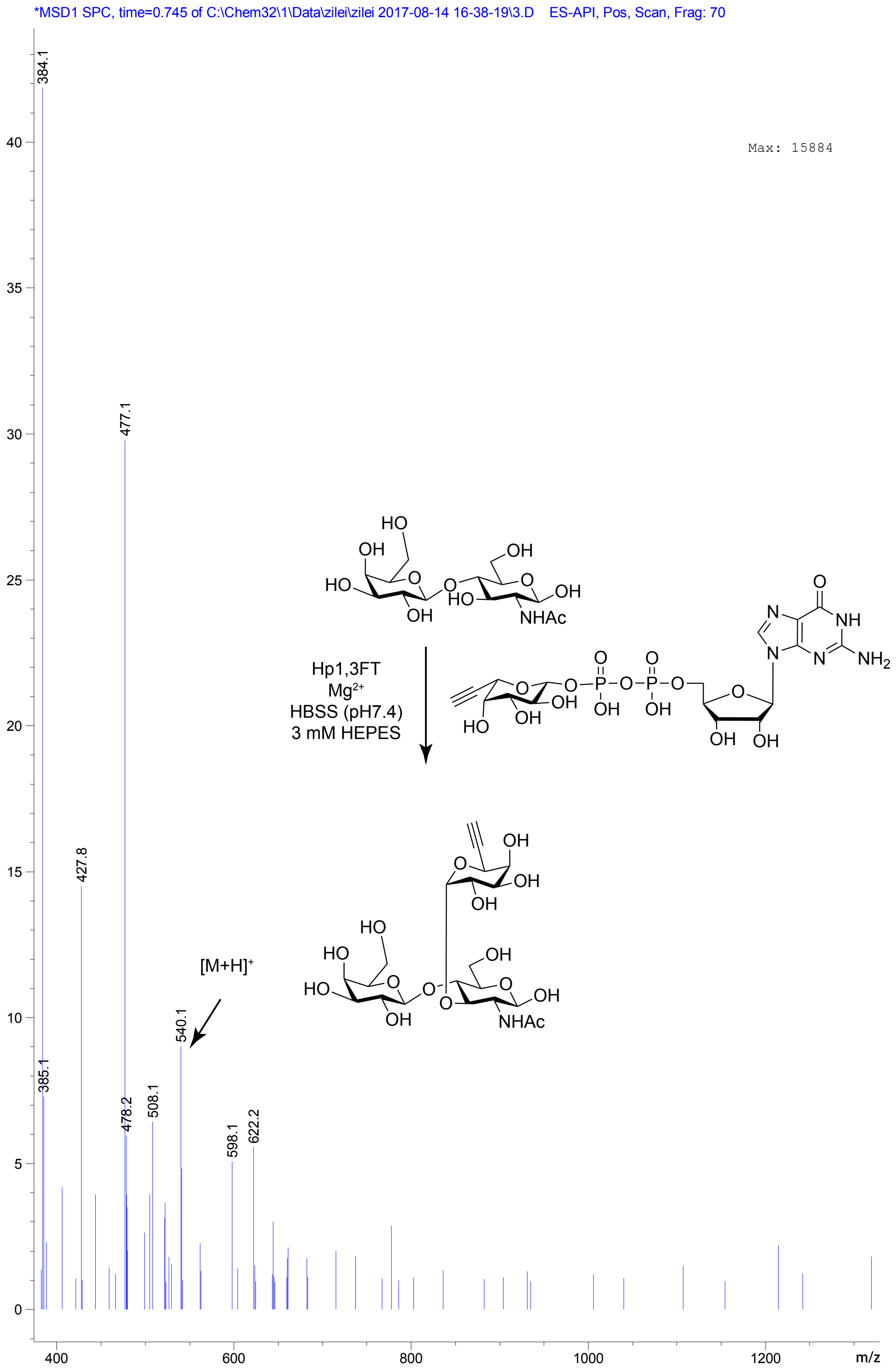

Sharpless 7/31/2018 3:22:41 PM SYSTEM Page IX of XI

Supplementary Figure 22. Mass spectral characterization of Le^{X} derivte Galβ1-4(FucAlα1-3)-GlcNAc via LC/MS.

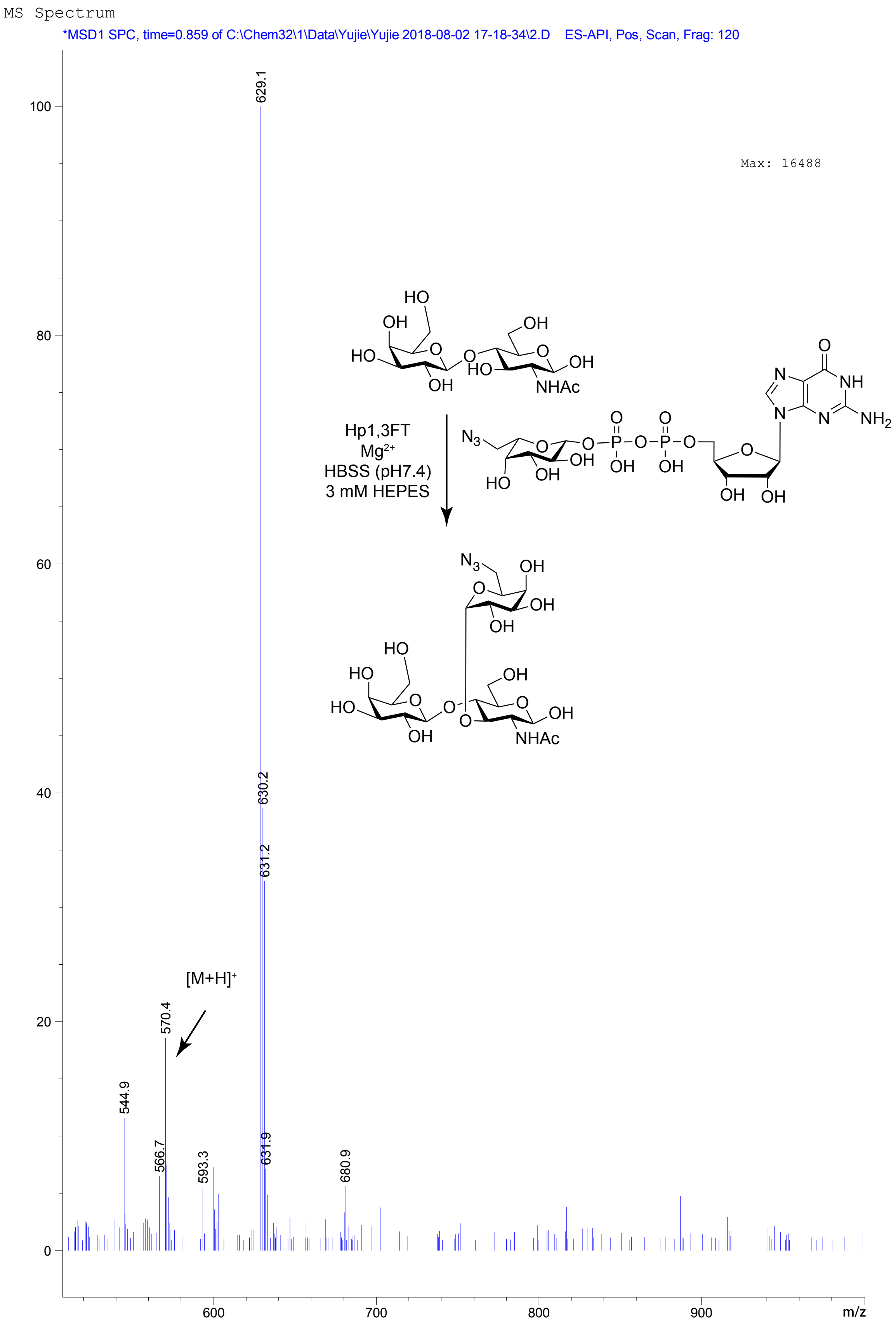

Supplementary Figure 23. Mass spectral characterization of Le^{X} derivte Galβ1-4(FucAzα1-3)-GlcNAc via LC/MS.

Print of window 80: MS Spectrum

MS Spectrum

*MSD1 SPC, time=1.073 of C:\Chem32\1\Data\SenlianH\SenlianH 2018-04-04 13-59-59\062-0301.D   ES-API, Pos, Scan, Frag:

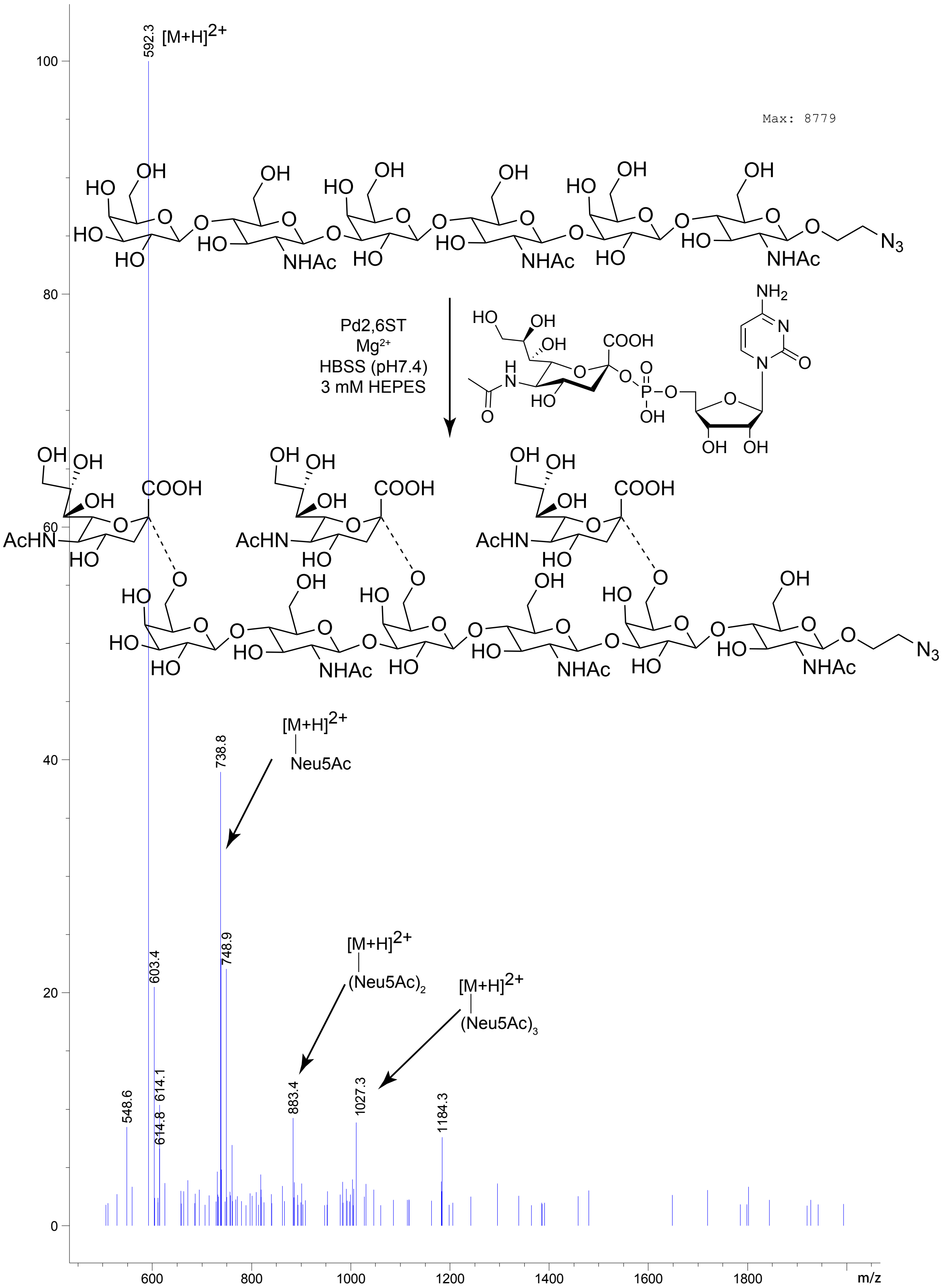

Sharpless 4/8/2018 2:43:48 PM SYSTEM   Page XI of XI

Supplementary Figure 24. LC/MS monitering the addition of α2-6-linked Neu5Ac onto the Gal of poly-(LacNAc)$_3$.

Supplementary Methods

General method and material: All commercialized chemical reagents utilized in this study were purchased from suppliers, and used without further purification unless noted. Alkynyl-PEG$_4$-biotin, Azide-PEG$_4$-biotin and Alkynyl-PEG-Cy3 were got from Click Chemistry Tools (Scottsdale, AZ, USA); Streptavidin-Alexa Fluor 488, 594 and 647 (AF488, AF594 and AF647) were obtained from Invitrogen (San Diego, CA, USA); Lectins (SNA, ECA, MAL, AAL and UEA) were purchased from Vector and used as instructed. Alexa Fluor 647-conjugated anti-human/mouse CLA antibody was purchased from Biolegend (cat. 321310). Horseradish peroxidase-conjugated anti-biotin antibody (HRP-anti-biotin antibody, 200-032-211) was purchased from Jackson ImmunoResearch Laboratories (West Grove, PA). The flow cytometry quantification was performed on Attune NxT flow cytometry (Life). The fluorescent images were taken on BZ-X700 (KEYENCE)

Synthesis of chemical compounds: BTTPS[1], GDP-fucose[2], GDP-FucAl[2], GDP-FucAz[2], CMP-NeuAc[3], CMP-SiaNAz[3], CMP-9AzSia[3], CMP-Sia*N*Al[4] and CMP-Sia*N*Poc[5] were synthesized as previously reported. For conjugation of dye (Cy3) or biotin onto GDP-fucose, we conducted the reaction of GDP-FucAz and Alkynyl-PEG$_4$-biotin (or Alkynyl-PEG-Cy3) via copper (I)-catalyzed azide-alkyne [3+2] cycloaddition. Briefly, GDP-FucAz (10 mM) was dissolved in water containing 10 mM Al-PEG$_4$-biotin (Click Chemistry Tools), 1 mM Cu_2SO_4 (ligand BTTPS and Cu^{2+} pre-mixture at molar ratio 2:1) and 2.5 mM sodium ascorbate. The reaction was maintained in 30 ^{o}C with gentle vortex (250 rpm) for 6 hours, and monitored by LC-MS using a negative MS mode. After reaction completion, the mixture was quenched with 2 mM bathocuproine disulfonate. This crude product was roughly considered as 10 mM GDP-FucAz/Al-PEG$_4$-biotin (or GDP-FucAz/Al-Cy3) conjugate and then titrated in live cell surface fucosylation and glycan labeling. Similarly, for the synthesis of CMP-Sia*N*Az/Al-PEG$_4$-biotin conjugate, CMP-Sia*N*Az (20 mM) were solved in water containing 20 mM Al-PEG$_4$-biotin (Click Chemistry Tools), 2 mM Cu_2SO_4 (ligand BTTPS and Cu^{2+} pre-mixture at molar ratio 2:1) and 2.5 mM sodium ascorbate. The reaction was maintained in 30 ^{o}C with gentle vortex (250 rpm) for 6 hours, and monitored by LC-MS using a negative MS mode. After reaction completion, the mixture was quenched with 4 mM bathocuproine disulfonate. This crude product was roughly considered as 20 mM CMP-Sia*N*Az/Al-PEG$_4$-biotin conjugate and then titrated in live cell surface on-step glycan labeling.

Cell culture: Chinese hamster ovary (CHO) cells, CHO-Lec 2 cells and CHO-Lec 8 cells were obtained from the American Type Culture Collection (Manassas, VA), and were rutinely kept in high-glucose DMEM medium supported with 10% (vol/vol) heat-inactivated FBS, 100 U/mL penicillin G, and 100 mg/mL streptomycin. Madin-Darby canine kidney (MDCK-SIAT1) cells were purchased from Sigma-Aldrich, and were cultureed in high-glucose DMEM medium supported with 10% (vol/vol) heat-inactivated FBS, 1 mg/mL G418, and 2 mM glutamine. Cells were incubated in a water-saturated, 5% CO_2 cultivator at 37°C, under the control of the absence of mycoplasma contamination.

Expression and purification of glycosyltransferases: Bacterial recombinant fucosyltransferases (Hm1,2FT and Hp1,3FT) and sialyltransferases (Pm2,3ST-M144D and Pd2,6ST) were purified according to a previously described procedure with several modifications. E. coli BL21(DE3) cells harboring the expression vectors for His$_6$-tagged Hm1,2FT, Hp1,3FT, Pm2,3ST- M144D, or Pd2,6ST were incubated in 1L LB broth with corresponding antibiotics (see Figure S1B) at 37°C until OD_{600}=0.7. The culture was then induced with 0.1mM isopropyl e incubated in 1L LB broth with corresponding antibiotics (se. After induction, the *E. coli* cells were harvested, suspended in 40 mL lysis buffer (20 mM Tris-HCl, pH7.5, 500 mM NaCl, 10 mM imidazole, 1 mM DTT), and lysed by sonication. Cell debris was pelleted by centrifuging at 8000 xg for 30 min, and 1 mL Ni-sepharose resin was added to the supernatant. After incubating at 4°C with rotation for 4 hours, the supernatant with resin was applied onto a Poly-Prep column and washed with >20mL wash buffer (20 mM Tris-HCl, pH 7.5, 500 mM NaCl, 50 mM imidazole, 0.5 mM DTT). The His$_6$-tagged enzymes were then eluted with ~12 mL elution buffer (2 mM Tris-HCl, pH 7.5, 500 mM NaCl, 500 mM imidazole, 0.5 mM DTT), concentrated, and changed to storage buffer using 50mL ultra centrifugal filters (10k Da molecular weight cut-off). The purified enzymes were confirmed by SDS-PAGE (Figure S1A), diluted to 1.0 mg/mL with storage buffer, and stored at -80 °C or 4 °C. Human recombinant STs, ST6Gal1 and ST3Gal4 were purified as previously reported.[6,7]

MALDI-TOF mass spectrometry profiling N-linked glycans: *N*-glycans from MDCK cells with cell-surface glycan edited or not, were analyzed by MALDI-TOF mass spectrometry via permethylation as previously reported.[8,9] Briefly, MDCK cells were collected for each sample about 5 x 10^7 cells. Cells were lysed with RIPA buffer after Pd2,6ST-assisted cell-surface glycan modification via 60 min-incubation at 37 ^{o}C in HBSS buffer (pH7.4) containg 3 mM HEPES, 20 mM $Mg2^{+}$, 50 μg/mL Pd2,6ST and 200 μM CMP-NeuAc. The control samples were cells without Pd2,6ST-assisted glycan modification, and directly lysed with RIPA buffer. The proteins in the super-

natant were precipitated with methanol and choloroform. Then, the pellets were dissolved in PBS (pH7.4) with 1% SDS. After dialysis, the samples were lyophilized and resuspend in 400 μL of 50 mM ammonium bicarbonate (pH 8.5). The reduction was performed by adding 20 μL of 250 mM dithiothreitol (DTT) and incubated at 50 ℃ for 1.5 h. The carboxymethylation was carried out by adding 40 μL of 450 mM iodoacetamide (IAM), and incubating in dark at room temperature for another 1.5 h. To remove DTT and IAM, the samples were dialyzed. The tryptic digest was performed by adding 5 μg of trypsin (Promega, V5111) to each sample, and incubating for 20 h at 37 °C. The digested sample was then heated for 10 min at 100℃ to inactivate the trypsin, and lyophilized. To purify the peptides, the reverse-phase C18 Sep-Pak cartridge (Waters, 1 cc) was primed sequentially with 5 mL methanol, 5 mL acetonitrile, 5mL isopropanol, and 15 mL 5% acetic acid. Then the peptides dissolved in 5% acetic acid was loaded onto the column, washed with ~10 mL of 5% acetic acid, and eluted with a gradient of 20% to 80% isopropanol. After drying with a Speed Vac concentrator, the eluted fractions were combined and resuspended in 200 μL 1x NEB Glycobuffer 2. The *N*-glycans were released by adding 8 μL of PNGaseF (NEB, P0704S), and incubated at 37 ℃ for 18 h. The digestion was terminated by lyophilization, and the released *N*-glycans were resuspended in 400 μL of 0.1% trifluoroacetic acid (TFA) and purified via a reverse-phase C18 Sep-Pak cartridge. The columns were sequentially conditioned with 5 mL methanol and 10 mL 0.1% TFA, before loading the samples. Then washed with 3 mL of 0.1% TFA, the flowthrough and wash fractions were collected and lyophilized on a SpeedVac concentrator. The purified *N*-glycans resuspending in 150 μL DMSO and 0.5 μL water, were permethylated by adding 150 μL of slurry (about 200 mg of NaOH was grinded with 1 mL of DMSO in a dry mortar to form a white slurry) and 150 μL of iodomethane. The tube space above the sample was filled with nitrogen gas before sealed, and incubated at room temperature in dark for 25 min with vigorous vortexing. The reaction was quenched by adding 300 μL water dropwise, and neutralized with 300~450 μL 1M HCl. The excess iodomethane was removed by pipetting air into the tube until all solid is dissolved and all air is gone. Permethylated glycans were extracted by dichloromethane (washed with water for several times and dried with a SpeedVac concentrator) for three to four times. To desalt the permethylated glycans, the dried samples were resuspended in 200 μL methanol, and loaded onto a C18 column that was sequentially conditioned with 5 mL methanol, 5 mL water, 5 mL acetonitrile and 5 mL water. The column was then sequentially washed with 15 mL water and 2 mL 10% acetonitrile, before the permethylated glycans were eluted with 3 mL of 80% acetonitrile and 1 mL 100% acetonitrile. The eluted fractions were further dried with a SpeedVac concentrator, before resuspended in 20 μL of 50% methanol. The glycans were then analyzed by MALDI-TOF: 2 μL sample were mixed with 2 μL 2,5-dihydroxybenzoic acid (DHB) matrix, and 1 μL mixture was spotted onto the target plate and air-dried. MALDI-TOF data was acquired on a 5800 MALDI-TOF AB SCIEX mass spectrometer in the positive, reflectron mode with molecular weight ranging from 1800~4600 Da.

Supplementary References

1. Wang, W. *et al.* Sulfated ligands for the copper(I)-catalyzed azide-alkyne cycloaddition. *Chem. Asian J.* **6,** 2796-2802 (2011).
2. Wang, W. *et al.* Chemoenzymatic synthesis of GDP-L-fucose and the Lewis X glycan derivatives. *Proc. Natl. Acad. Sci. U.S.A.* **106,** 16096-16101 (2009).
3. Yu, H., Yu, H., Karpel, R. & Chen, X. Chemoenzymatic synthesis of CMP-sialic acid derivatives by a one-pot two-enzyme system: comparison of substrate flexibility of three microbial CMP-sialic acid synthetases. *Bioorg. Med. Chem.* **12,** 6427-6435 (2004).
4. Noel, M. *et al.* Probing the CMP-Sialic Acid Donor Specificity of Two Human β-d-Galactoside Sialyltransferases (ST3Gal I and ST6Gal I) Selectively Acting on O- and N-Glycosylproteins. *Chembiochem* **18,** 1251-1259 (2017).
5. Briard, J. G., Jiang, H., Moremen, K. W., Macauley, M. S. & Wu, P. Cell-based glycan arrays for probing glycan-glycan binding protein interactions. *Nat. Commun.* **9,** 880-890 (2018).
6. Jiang, H. *et al.* Modulating Cell-Surface Receptor Signaling and Ion Channel Functions by In Situ Glycan Editing. *Angew. Chem. Int. Ed. Engl.* **130,** 979-983 (2018).
7. Mbua, N. E. *et al.* Selective Exo-Enzymatic Labeling of N-Glycans on the Surface of Living Cells by Recombinant ST6Gal I. *Angew. Chem. Int. Ed. Engl.* **52,** 13012-13015 (2013).
8. Ciucanu, I. & Costello, C. E. Elimination of oxidative degradation during the per-O-methylation of carbohydrates. *J. Am. Chem. Soc.* **125,** 16213-16219 (2003).
9. North, S. J. *et al.* Glycomics profiling of Chinese hamster ovary cell glycosylation mutants reveal N-glycans of a novel size and complexity. *J. Biol. Chem.* **285,** 5759-5775 (2010).
