# Supplementary material for: Bacterial glycosyltransferase-mediated cell-surface chemoenzymatic glycan modification
Source: Nat Commun. 2019 Apr 17;10:1799. doi: 10.1038/s41467-019-09608-w (PMC6470217; doi:10.1038/s41467-019-09608-w)
Supplement: Supplementary file 2 — Reporting Summary [file 41467_2019_9608_MOESM2_ESM.pdf]

nature research

Corresponding author(s): Peng Wu

Last updated by author(s): Feb 12, 2019

Reporting Summary

Nature Research wishes to improve the reproducibility of the work that we publish. This form provides structure for consistency and transparency in reporting. For further information on Nature Research policies, see Authors & Referees and the Editorial Policy Checklist.

Statistics

For all statistical analyses, confirm that the following items are present in the figure legend, table legend, main text, or Methods section.

n/a	Confirmed	
☐	☒	The exact sample size (*n*) for each experimental group/condition, given as a discrete number and unit of measurement
☐	☒	A statement on whether measurements were taken from distinct samples or whether the same sample was measured repeatedly
☐	☒	The statistical test(s) used AND whether they are one- or two-sided<br>*Only common tests should be described solely by name; describe more complex techniques in the Methods section.*
☒	☐	A description of all covariates tested
☒	☐	A description of any assumptions or corrections, such as tests of normality and adjustment for multiple comparisons
☐	☒	A full description of the statistical parameters including central tendency (e.g. means) or other basic estimates (e.g. regression coefficient) AND variation (e.g. standard deviation) or associated estimates of uncertainty (e.g. confidence intervals)
☐	☒	For null hypothesis testing, the test statistic (e.g. *F*, *t*, *r*) with confidence intervals, effect sizes, degrees of freedom and *P* value noted<br>*Give P values as exact values whenever suitable.*
☒	☐	For Bayesian analysis, information on the choice of priors and Markov chain Monte Carlo settings
☒	☐	For hierarchical and complex designs, identification of the appropriate level for tests and full reporting of outcomes
☒	☐	Estimates of effect sizes (e.g. Cohen's *d*, Pearson's *r*), indicating how they were calculated

Our web collection on statistics for biologists contains articles on many of the points above.

Software and code

Policy information about availability of computer code

Data collection	n/a
Data analysis	n/a

For manuscripts utilizing custom algorithms or software that are central to the research but not yet described in published literature, software must be made available to editors/reviewers. We strongly encourage code deposition in a community repository (e.g. GitHub). See the Nature Research guidelines for submitting code & software for further information.

Data

Policy information about availability of data

All manuscripts must include a data availability statement. This statement should provide the following information, where applicable:

- Accession codes, unique identifiers, or web links for publicly available datasets
- A list of figures that have associated raw data
- A description of any restrictions on data availability

Data and software availability: The binding of sLeX with HK68-HA and HK68-MTA-HA was modeled based on aligning the corresponding HA apo structures to the crystal structure of A/canine/Colorado/17864/06 (H3 subtype) HA in complex with sLeX (by PyMOL11). Structure information resources, HK68-HA (PDB 4FNK), HK68-MTA-HA (PDB 5VTX), canine HA13. Alignment was performed using the receptor-binding subdomain (residues 117 to 265 of HA1). The raw data that support the findings of this study are available from the authors on reasonable request.

Field-specific reporting

Please select the one below that is the best fit for your research. If you are not sure, read the appropriate sections before making your selection.

☒ Life sciences ☐ Behavioural & social sciences ☐ Ecological, evolutionary & environmental sciences

For a reference copy of the document with all sections, see nature.com/documents/nr-reporting-summary-flat.pdf

Life sciences study design

All studies must disclose on these points even when the disclosure is negative.

Sample size	for the chemoenzymatic labeling assays, the sample size is 3 biological repeats; for the influenza A virus-direct host cell killing assays, the sample size is 6 biological repeats.
Data exclusions	for the influenza A virus caused host cell killing assays, the contaminated repeats were excluded from data assay
Replication	3 to 6 were performed in our study
Randomization	n/a
Blinding	n/a

Reporting for specific materials, systems and methods

We require information from authors about some types of materials, experimental systems and methods used in many studies. Here, indicate whether each material, system or method listed is relevant to your study. If you are not sure if a list item applies to your research, read the appropriate section before selecting a response.

Materials & experimental systems

n/a	Involved in the study	
☐	☒	Antibodies
☐	☒	Eukaryotic cell lines
☒	☐	Palaeontology
☒	☐	Animals and other organisms
☒	☐	Human research participants
☒	☐	Clinical data

Methods

n/a	Involved in the study	
☒	☐	ChIP-seq
☐	☒	Flow cytometry
☒	☐	MRI-based neuroimaging

Antibodies

Antibodies used	anti-biotin; anti-human/mouse CLA
Validation	n/a

Eukaryotic cell lines

Policy information about cell lines

Cell line source(s)	CHO, CHO-Lec2 and CHO-Lec8 were obtained from ATCC; MDCK-SIAT1 was purchased from Sigma-Aldrich
Authentication	All cell lines were purchased from commercial suppliers and directly used without further authentication.
Mycoplasma contamination	not detected
Commonly misidentified lines (See ICLAC register)	n/a

Flow Cytometry

Plots

Confirm that:

☒ The axis labels state the marker and fluorochrome used (e.g. CD4-FITC).

☒ The axis scales are clearly visible. Include numbers along axes only for bottom left plot of group (a 'group' is an analysis of identical markers).

☒ All plots are contour plots with outliers or pseudocolor plots.

☒ A numerical value for number of cells or percentage (with statistics) is provided.

Methodology

Sample preparation	The cultured cells were collected, labeled chemoenzymatically, or stained with antibody/lectin and then analyzed.
Instrument	Attune NxT flow cytometry (Life)
Software	FlowJo v10.1r5
Cell population abundance	Gated cells from all samples were between 70~90% of all events.
Gating strategy	The starting cell populations were gated from FSC/SSC plot.

☐ Tick this box to confirm that a figure exemplifying the gating strategy is provided in the Supplementary Information.
